# Encoding of Spatio-Temporal Input Characteristics by a CA1 Pyramidal Neuron Model

**DOI:** 10.1371/journal.pcbi.1001038

**Published:** 2010-12-16

**Authors:** Eleftheria Kyriaki Pissadaki, Kyriaki Sidiropoulou, Martin Reczko, Panayiota Poirazi

**Affiliations:** 1 Department of Biology, University of Crete, Heraklion, Crete, Greece; 2 Institute of Molecular Biology and Biotechnology, Foundation for Research and Technology, Heraklion, Crete, Greece; 3 Institute of Molecular Oncology, Alexander Fleming Biomedical Sciences Research Center, Athens, Greece; Université Paris Descartes, Centre National de la Recherche Scientifique, France

## Abstract

The *in vivo* activity of CA1 pyramidal neurons alternates between regular spiking and bursting, but how these changes affect information processing remains unclear. Using a detailed CA1 pyramidal neuron model, we investigate how timing and spatial arrangement variations in synaptic inputs to the distal and proximal dendritic layers influence the information content of model responses. We find that the temporal delay between activation of the two layers acts as a switch between excitability modes: short delays induce bursting while long delays decrease firing. For long delays, the average firing frequency of the model response discriminates spatially clustered from diffused inputs to the distal dendritic tree. For short delays, the onset latency and inter-spike-interval succession of model responses can accurately classify input signals as temporally close or distant and spatially clustered or diffused across different stimulation protocols. These findings suggest that a CA1 pyramidal neuron may be capable of encoding and transmitting presynaptic spatiotemporal information about the activity of the entorhinal cortex-hippocampal network to higher brain regions via the selective use of either a temporal or a rate code.

## Author Summary

Pyramidal neurons in the hippocampus are crucially involved in learning and memory functions, but the ways in which they contribute to the processing of sensory inputs and their internal representation remain mostly unclear. The principal neurons of the CA1 region of the hippocampus are surrounded by at least 21 different types of interneurons. This feature, together with the fact that CA1 pyramidal dendrites associate two major glutamatergic inputs arriving from the entorhinal cortex, makes it laborious to track the ‘how’ and ‘what’ of synaptic integration. The present study tries to shed light on the ‘what’, that is, the information content of the CA1 discharge pattern. Using a detailed biophysical CA1 neuron model, we show that the output of the model neuron contains spatial and temporal features of the incoming synaptic input. This information lies in the temporal pattern of the inter-spike intervals produced during the bursting activity which is induced by the temporal coincidence of the two activated synaptic streams. Our findings suggest that CA1 pyramidal neurons may be capable of capturing features of the ongoing network activity via the use of a temporal code for information transfer.

## Introduction

The Cornus Ammonis 1 (CA1) region serves as the output of the hippocampus and has been associated with several memory processes including working memory [1], [2], [3], [4], acquisition and retrieval of contextual fear conditioning [4] as well as spatial and object novelty detection [5], [6], [7]. Therefore, understanding information coding in CA1 neurons is critical for the interpretation of hippocampal output and its possible role in these memory processes.

CA1 pyramidal neurons receive sensory information via the perforant path (PP) or temporoammonic (TA) pathway from the Entorhinal Cortex (EC) and processed intra-hippocampal input from CA3 neurons through the Schaffer Collaterals (SC) pathway. The interplay between EC and CA3 signals has been shown to modulate the discharge pattern of CA1 pyramidal neurons in a bi-directional way [8], [9], [10], [11], [12], [13], [14]: for example, when the TA pathway is activated a few hundreds of milliseconds before the SC pathway, the propagation of supratheshold SC signals is blocked [12]; on the contrary, when the TA pathway is activated a few tens of milliseconds before the SC pathway, the propagation of subthreshold SC signals is facilitated [9]. This negative or positive modulation of excitability may be attributed to the domination of either the inhibitory (GABAergic) or the excitatory component of TA-mediated inputs and has been suggested to act as a gating mechanism [10]. It has also been reported that inhibition modulates action potential firing rate by blocking dendritic calcium activity [15].

In addition to pathway-specific interactions, the dendritic arrangement of activated synapses has also been shown to influence neuronal output. Both experimental and computational studies suggest that activation of synapses in clusters within a few dendritic compartments leads to much stronger somatic responses than activation of diffusely arranged synapses [16], [17], [18], [19], [20]. This arrangement-dependent modulation of excitability has been associated with the formation of long-term memory engrams [21], the transfer of spatial information from EC to CA1 [22], the binding of behaviorally linked information [23] and has given rise to the ‘branch strength potentiation’ as a new plasticity mechanism for storing complex characteristics of the synaptic input [24]. Finally, results from the barn owl auditory system provide one of the first direct evidence about the clustering of axo-dendritic contacts in response to behaviorally relevant learning signals [25].

Since hippocampal firing patterns are known to vary depending on the ongoing memory process and the behavioral state of the animal [26], [27], [28], [29], the interplay between lamina-specific signals, which can alter the response of pyramidal neurons from regular spiking to bursting or activity blockade, is likely to play a key role in information processing in the hippocampus. In this work, we use a detailed compartmental model of a CA1 pyramidal neuron to investigate the possibility that the interplay between extra-hippocampal (EC) and intra-hippocampal (CA3) signals that vary in their spatio-temporal characteristics may serve as a substrate for information coding.

## Results

**Figure 1:**
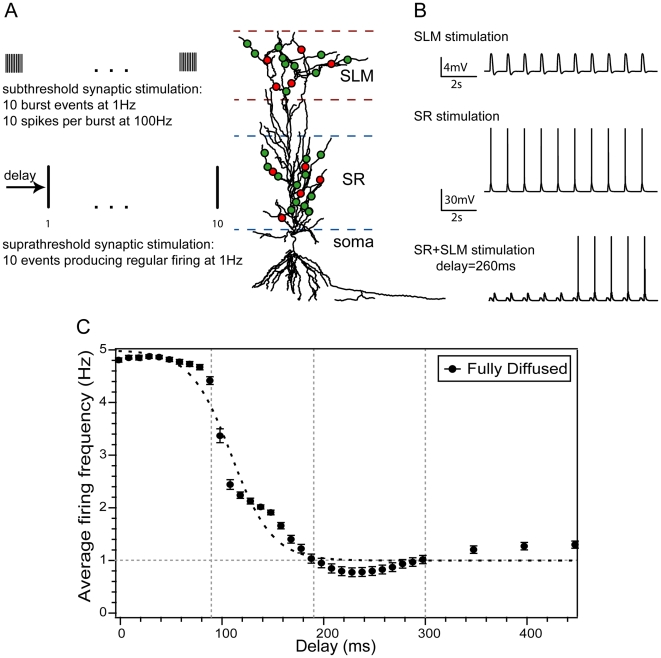
Graphic representation of the initial stimulation protocol. A. Synaptic contacts were distributed in two layers corresponding to the Stratum Radiatum (SR) and Stratum Lacunosum Moleculare (SLM). Both excitatory (green) and inhibitory (red) synapses were placed in each layer. Synapses in the SLM layer were activated by high frequency subthreshold bursts (10 spikes at 100 Hz) for 10 seconds, at a frequency of 1 Hz. Synapses in the SR layer were activated by suprathreshold events strong enough to induce on average a single somatic AP at a frequency of 1 Hz. Stimulation of the two pathways was separated in time by a delay raging from 0–450 ms (0–0.45 s), with SLM stimulation preceding the SR input. B. Somatic traces showing the response of the model to stimulation of the SLM layer alone, the SR layer alone and both layers with a delay of 260 ms (0.26 s). Traces correspond to the fully diffused synaptic arrangement. For a delay of 260 ms (0.26 s), somatic spikes are truncated for the first 5 seconds. C. Average firing frequency as a function of the stimulus delay for the fully diffused synaptic arrangement. Short delays (0–90 ms, (0–0.09 s)) are associated with pronounced enhancement of the neuronal firing frequency while long delays (190–350 ms, (0.19–0.35 s)) lead to suppression of excitability (*ff* drops below the 1 Hz baseline). Overall, the average firing frequency of the model is modulated by the temporal delay between incoming signals in a sigmoidal shaped pattern. Error bars represent standard error.

In order to investigate the effect of temporal and spatial variability of lamina-specific inputs on the firing properties of CA1 pyramidal neurons we simulate synaptic stimulation of the Stratum Radiatum (SR) and Stratum Lacunosum Moleculare (SLM) layers (0–290 and 385–470 microns, (0–2.9·10^−4^ and 3.85–4.7·10^−4^ m) from the soma, respectively) in a morphologically and biophysically detailed model cell (see Methods). We use 84% excitatory and 16% inhibitory synapses in the SLM layer and 75% excitatory and 25% inhibitory in the SR layer, according to the anatomical data of [30]. Distal dendrites of CA1 neurons in the SLM layer predominately receive GABAergic input from neurogliaform (NG) interneurons[31], [32] and perforant path-associated cells [31] while the SR apical trunk receives inhibitory input mostly from apical dendrite innervating cells [33] [34], PV and somatostatin positive bistratified cells [35] and ivy cells [36]. NG interneurons in young animals exhibit GABA_B_-dependent depression of EPSCs after high-frequency stimulation of the perforant path and respond with high frequency bursts when activated by strong current pulses [32]. Parvalbumin-containing neurons (basket cells) innervating the proximal dendritic and somatic regions of CA1 neurons generate primarily fast IPSPs characteristic of GABA_A_ receptors and follow pyramidal cell discharges by a monosynaptic delay both *in vitro* [37] and *in vivo* [38]. We use the stimulation protocol shown in Figure 1A, which was first described by Dvorak-Carbone and Schuman [12] and takes into account the main activation patterns of inhibitory as well as excitatory input to CA1 pyramidal neurons. According to this protocol, excitatory and inhibitory synapses in the SLM layer are stimulated with subthreshold bursts (each burst contains 10 events at 100 Hz and leads to ∼2 mV depolarization) delivered at a frequency of 1 Hz for 10 seconds. Excitatory and inhibitory synapses in the SR layer receive 1 Hz stimulation (1 event per second, for 10 seconds) capable of inducing regular spiking at 1–3 Hz for 10 seconds. In our set of experiments, the two layers are stimulated with 34 different delays ranging from 0–450 ms (0–0.45 s), with the SLM layer being activated first, and four different synaptic arrangements (fully diffused, fully clustered, SR clustered and SLM clustered) in which synapses are either randomly scattered throughout each layer or clustered within a few branches. Both excitatory (containing AMPA and NMDA receptors) and inhibitory (containing GABA_A_ or GABA_B_ receptors) synapses are used in the SR and SLM layers as described in the Methods section. For each different combination of temporal delay and type of synaptic arrangement, we perform 100 trials in which the exact synapse locations vary at random, resulting in a total of 13,600 simulated firing patterns. Representative model responses to SLM, SR and SLM+SR stimulation recorded at the cell body are shown in Figure 1B.

### The SR delay acts as a temporal switch between neuronal excitability modes

To examine the effect of delayed and layer-specific synaptic activation on the excitability of the model neuron, we record its average firing frequency (*ff*) for the fully diffused arrangement across the 34 different delays. For each delay, *ff* is calculated over 62 repetition trials (see Methods). As shown in Figure 1C, the average *ff* of the model neuron is strongly modulated by the delay between the two stimulated layers and this modulation can be described by a sigmoidal activation function (see Table 1, F_Exp_1_). For short delays (0–90 ms (0–0.09 s)), *ff* is clearly greater than baseline (1 Hz), for intermediate delays (100–190 ms (0.1–0.19 s)), *ff* falls off exponentially, and for long delays (200–300 ms (0.2–0.3 s)), *ff* remains below the baseline exhibiting the ‘spike blocking’ phenomenon, in accordance with previous work [12], [39] (also see Figure S6 in Text S1). These results suggest that the effect of preceding SLM stimulation on low frequency SR-induced activity depends heavily on the delay of SR stimulation and changes from facilitation (short delays) to suppression (long delays) according to a sigmoidal function. A similar facilitation of the model's somatic responses, associated with the generation of dendritic plateau potentials, was also shown using a theta-burst stimulation protocol (see Figure 3 in [40]) and was recently verified experimentally by [41]. Taken together, these results show that the response of the single neuron model to a simple protocol can replicate findings from different experimental groups and different experimental conditions, including both the facilitation and suppression of CA1 neuronal activity depending on the temporal delay between SR and SLM signals [9], [12], [41].

**Table 1:** Sigmoidal equations used to fit the average firing frequency curves in Figures 1–2.

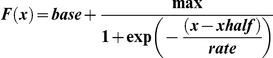	F_Exp_1_(x)±s.d.	F_Exp_2_(x) ±s.d.	F_Exp_3_(x) ±s.d.	F_Exp_4_(x) ±s.d.
base	4.964±0.117	4.7927±0.0522	4.7907±0.046	4.4994±0.091
max	−3.962±0.142	−2.961±0.0652	−2.850±0.0616	−3.9574±0.122
xhalf	111.31±2.87	120.23±1.79	114.77±1.45	122.12±2.54
rate	20.152±2.5	19.131±1.57	12.603±1.26	24.834±2.28

**Figure 2:**
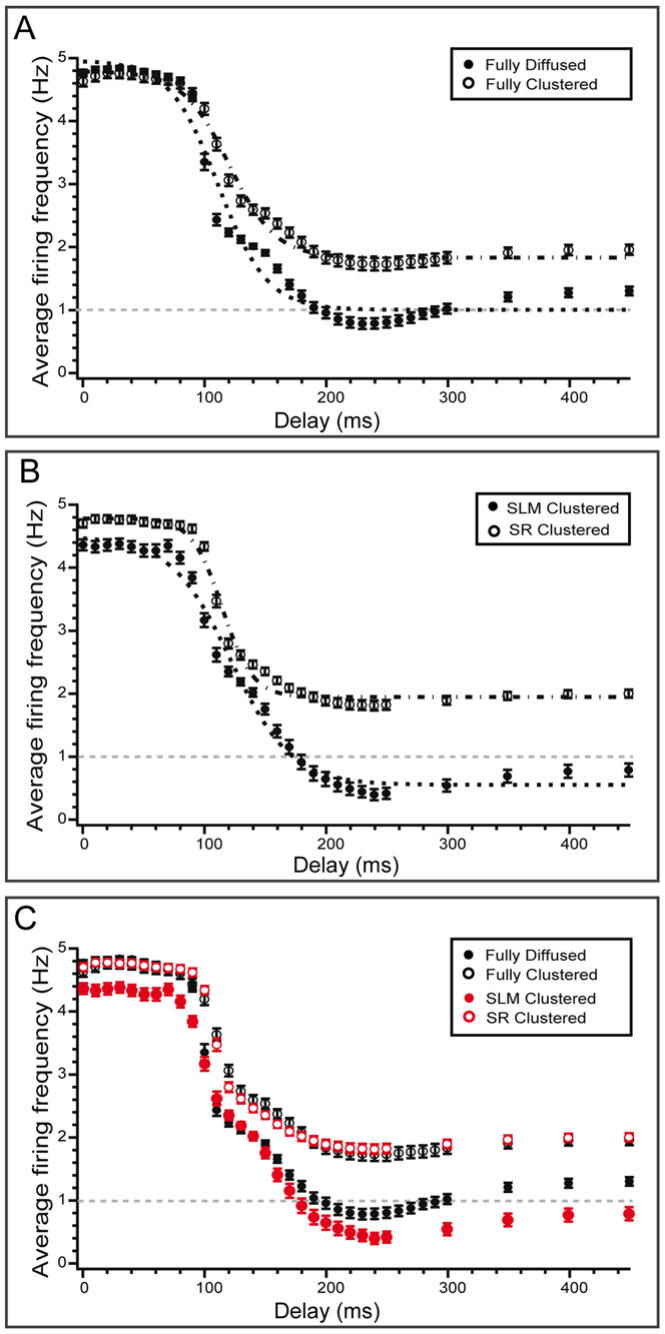
Average firing frequency as a function of the temporal delay for all different synaptic arrangements. A. Synaptic clustering in both layers (filled circles) eliminates the excitability suppression effect that was evident for long delays in the diffused arrangement (open circles). However, model responses for short delays (0–90 ms, (0–0.09 s)) are nearly identical for both arrangements. Overall, synaptic clustering in both layers results in enhancement of the average firing frequency of the model for all delays larger than 100 ms (0.1 s). B. Layer specific synaptic clustering has different effects on neuronal output. Clustering in the SLM layer enhances and prolongs excitability suppression for delays larger than 190 ms. Moreover, the average firing frequency in the SLM clustered case (panel B, filled circles) is lower than the fully diffused case (panel A, filled circles) for all delays. Clustering in the SR layer has a very similar effect as clustering in both layers: excitability is enhanced across all delays. C. Comparison of average *ff* for all arrangements. Clustering in the SR is nearly identical to clustering in both layers (open circles) while clustering in the SLM results in reduction of the average *ff* across all delays and expansion of the spike blocking window. Error bars represent standard error.

To examine whether the excitability modulation exerted by the SR delay is affected by the spatial distribution of synaptic contacts, we repeat the experiment for all four synaptic arrangements and find a similar sigmoidal shaped modulation (Figure 2 and Table 1). The average firing frequency as a function of the temporal delay for the fully diffused (Exp1, filled circles) and fully clustered (Exp2, open circles) arrangements are shown in Figure 2A. For short delays (0–90 ms (0–0.09 s)) both arrangements result in a similar increase in excitability (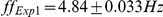, 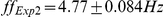) while for intermediate (100–190 ms, (0.1–0.19 s)) as well as longer delays (200–300 ms (0.2–0.3 s)) the average firing frequency of the model is significantly different between the two experiments (see Table S1 in Text S1). Moreover, in the fully clustered case, *ff* remains well above the baseline for all temporal delays, suggesting that the spike blocking phenomenon does not occur. Note that for delays longer than 160 ms, the average firing frequency of the model cell is clearly different for clustered versus diffused arrangements.

To determine which layer-specific synaptic arrangement may be responsible for the differences seen between Exp1 and Exp2, in the next simulation (Exp3) synapses are clustered only in the SR layer, while in Exp4 synapses are clustered only in the SLM layer. We find that the average firing frequency of the model in Exp3 and Exp4 is significantly different across all temporal delays (see Table S1 in Text S1). Moreover, model responses for the SR clustered case are almost identical to the responses for the fully clustered arrangement: *ff* is above baseline (no spike blocking) for all temporal delays in both arrangements (Figure 2A and 2B, open circles) and there is no statistical difference between the two curves (Figure 2C, open circles and Table S1 in Text S1). On the contrary, clustering in the SLM results in a general reduction of the average firing frequency across all delays and leads to more than 150 ms (0.15 s) prolongation of the excitability suppression period, thus qualitatively resembling the fully diffused experiment (Figure 2A, 2B and 2C filled circles).

Taken together, these findings suggest that the latency between incoming SR and SLM signals may determine the excitability level of a CA1 pyramidal neuron and serve as a signal for alternating between enhancement and suppression modes. Synaptic arrangement on the other hand seems to fine-tune this modulation of excitability by determining the degree of activity enhancement or suppression: clustering in the SR prevents spike blocking for long delays while clustering in the SLM reinforces spike blocking. The former suggests that clustered activation of SR synapses ensures the propagation of intra-hippocampal signals irrespectively of the timing and arrangement of SLM (i.e. cortical) input. The latter suggests that clustered activation of SLM synapses preferentially gates the propagation of delayed SR signals (i.e. intra-hippocampal input arriving after 200 ms (0.2 s)). Moreover, given the specific stimulation conditions, our model predicts that clustered input to the distal dendrites of the CA1 pyramidal neuron may act as an *AND* gate for short delays and as an *AND-NOT* gate for long delays [42].

**Figure 3:**
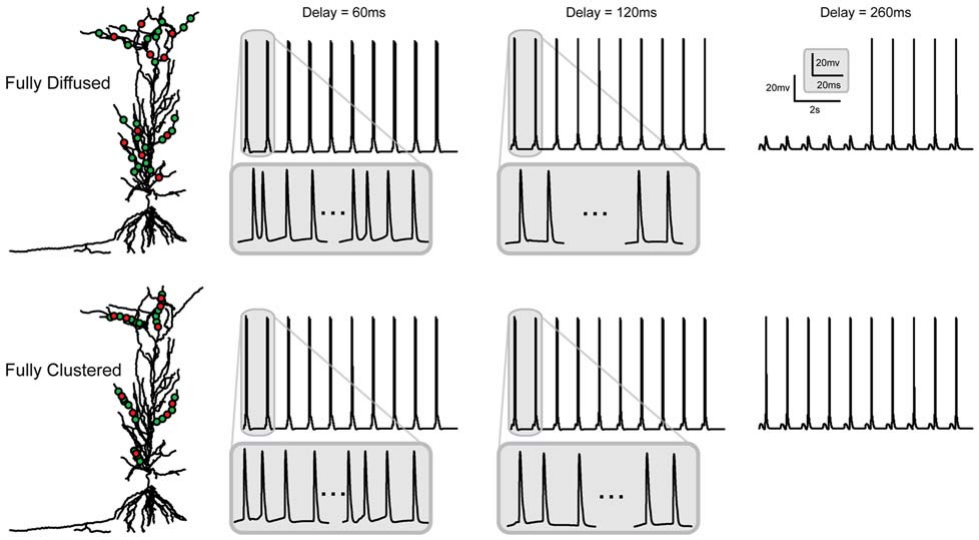
Representative traces showing the neuron's discharge pattern in various synaptic and temporal settings. Traces correspond to fully diffused and fully clustered synaptic arrangements and delays of 60, 120 and 260 ms (0.06, 0.12, 0.26 s), recorded at the cell body of the model cell. For a 60 ms (0.06 s) delay, strong somatic bursting is evident in both arrangements (inset: 4 spikes per event). As the delay increases to 120 ms (0.12 s), the bursting response weakens (inset: 2–3 spikes per event). For a delay of 260 ms (0.26 s), somatic spiking is partially blocked in the fully diffused arrangement while in the fully clustered arrangement the baseline response is unaffected.

Since both spatial and temporal features of the input affect the average firing frequency of the model neuron, we next investigate whether these changes are consistently reflected in the output pattern of the model cell. We find that for all synaptic arrangements and for temporal delays between 0 and 240 ms (0.24 s), the neuron often discharges with trains of bursts consisting of 2–6 action potentials and each burst appears with a frequency of 1 Hz, as dictated by the SR stimulation. In all four experiments, bursting is more prominent for short temporal delays (0–90 ms (0–0.09 s): 4–5 spikes per burst. 90–150 ms, (0.09–0.15 s): 2–3 spikes per burst. 160–240 ms (0.16–0.24 s): 1–2 spikes per burst). Representative traces for the fully clustered and fully diffused synaptic arrangements and three different delays (60, 120 and 260 ms (0.06, 0.12 and 0.26 s)) are shown in Figure 3. Note that while spike blocking is frequently evident for delays beyond 190 ms (0.19 s) in the fully diffused and SLM diffused arrangements, it is not unusual to find doublets in some somatic responses where spike blocking does not occur. These findings show that enhancement of the average firing frequency of the neuron results from the induction of a somatic bursting response which is affected by the spatio-temporal characteristics of the input.

### Biophysical mechanisms supporting bursting vs. spike blocking in the model cell

**Figure 4:**
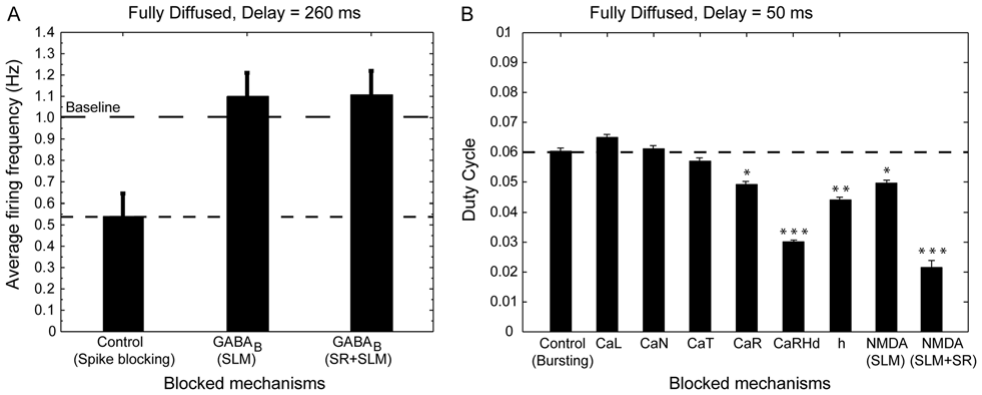
Biophysical mechanisms involved in excitability modulation. A. Effect of GABA_B_ blockade on the average firing frequency of the model cell during spike blocking conditions (fully diffused arrangement, delay = 260 ms, (0.26 s)). The average firing frequency of the model during spike blocking (control) is significantly below baseline. Blockade of the GABA_B_ receptor conductance by 90% either in the SLM layer or both SLM and SR layers restores baseline firing (1 Hz). B. Effect of blockade of different ionic mechanisms on the average duty cycle of the model cell during excitability enhancement conditions when the two pathways are stimulated with a delay of 50 ms (0.05 s) and the stimulated synapses are arranged in a diffused manner. The duty cycle [117] is used as a measure of ‘burstiness’, which is essentially the ratio of the active phase (burst duration) over the inter-burst-period (time from first spike of a burst until the first spike on the next burst). Blockade of the L-type, N-type and T-type calcium currents does not have a significant effect on the duty cycle while blockade of R-type calcium currents results in a small decrease of bursting (15.3% decrease) and blockade of the dendritic, high-threshold R-type channels (caRHd) reduces bursting up to 49.8%. Blockade of the h-current induces a 25% decrease in the duty cycle compared to the control. Blockade of the NMDA receptors in the SLM layer alone results in a 15% decrease of the duty cycle while blockade in both layers results in a 65% decrease in the duty cycle.

Previous experimental findings [12], [15], [43] suggested that distal dendritic inhibitory currents, primarily GABA_B_-mediated, selectively dampen the response of pyramidal neurons. To confirm that the GABA_B_ current is also necessary for the SLM-mediated suppression of excitability in our model neuron, we decrease the GABA_B_ current either in the distal dendrites or in all dendrites and record the model's response to spatially and temporally dispersed signals for which spike blocking is maximized: a fully diffused synaptic arrangement with 260 ms (0.26 s) delay between SLM and SR signals. Figure 4A shows that reduction of GABA_B_ -mediated currents by 90% in the SLM layer alone completely eliminates spike blocking (average firing frequency returns to baseline), reproducing the experimental data of [12], and further suggesting that GABA_B_ currents in the distal dendrites alone are primarily responsible for the spike blocking effect.

Somatic bursting observed for short delays in all different spatial arrangement experiments, is the flip-side of the coin regarding the EC-mediated modulation. Busting in pyramidal neurons has frequently been associated with the occurrence of dendritic regenerative events mediated primarily by Ca^++^ [41], [43], [44] and/or NMDA currents [41], [45], [46]. To investigate the biophysical mechanisms underlying burst generation in the model cell, we reduce the L-type, N-type, R-type and T-type calcium currents, the h-current and the NMDAR-mediated current by 90% and measure the effect on bursting (see Methods). Figure 4B, shows that blockade of L-type, N-type or T-type calcium currents does not significantly affect bursting while blockade of R-type calcium currents results in a small decrease of bursting (15.3% decrease) and blockade of the dendritic, high-threshold R-type channels (caRHd) reduces bursting up to 49.8%. Decreased bursting following a 90% elimination of R-type and R-type high threshold calcium conductances are in accordance with properties of the channels to drive after depolarization potentials and contribute to burst firing [47]. Blockade of the h-current also decreases bursting by 25%, probably due to the reduction in I_h_-mediated membrane depolarization following hyperpolarizing potentials. The latter negatively affects dendritic excitability and calcium spike initiation associated with somatic bursting [48], [49]. Blockade of the NMDA current in the SLM layer alone has a smaller impact (15% decrease) while blockade in both layers results in a remarkable 65% decrease in bursting. These findings suggest that NMDA-mediated currents along with R-type currents are the key players underlying somatic bursting, in accordance with the recent findings of [41]. Overall, our results add to a large body of previous work regarding the role of NMDA and voltage dependent calcium channels in promoting somatic bursting [41], [50], [51], [52], [53], [54], [55], [56].

### The spatio-temporal information content of bursts

#### The average burst inter-spike-interval

To further investigate whether spatio-temporal information of the incoming signals may be captured by the bursting activity of the model neuron, we measure the average Inter-Spike-Interval within bursts (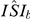) for all synaptic arrangements and delays between 0–240 ms (0–0.24 s) (see Methods). Only trials exhibiting bursting activity are used (at least two spikes per burst). Longer delays are not considered in this analysis since bursting is rarely observed in the fully diffused and SLM clustered cases.

**Figure 5:**
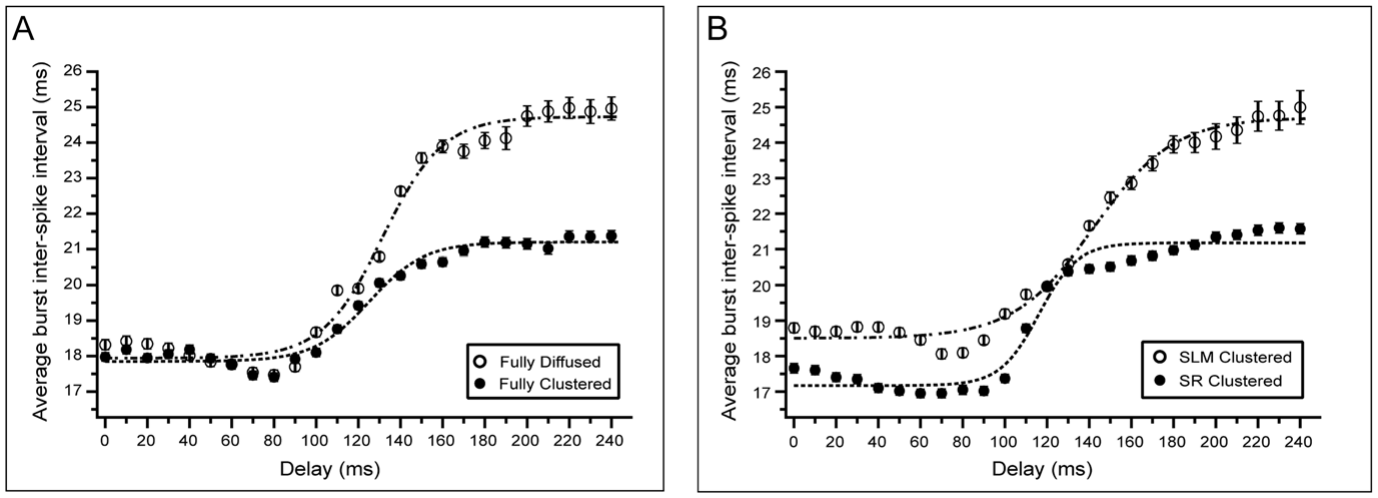
Average burst Inter-Spike-Interval (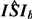) as a function of the temporal delay for all synaptic arrangements. Model responses can be approximated by sigmoidal functions. A. Synaptic clustering (filled circles) or diffusion (open circles) in both layers induce nearly identical average responses for short temporal delays (<100 ms) which become clearly different for large delays (>140 ms). B. Layer-specific synaptic clustering leads to separable 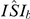 curves for most delays (except for 120–140 ms, (0.12–0.14 s)). Synaptic clustering in the SLM (open circles) seems to be primarily responsible for changes in the 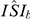 values corresponding to inputs with short temporal delays. Error bars represent standard error.

Similar to the average firing frequency, the 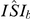 values for both fully clustered and fully diffused arrangements are nearly identical for short delays (0–90 ms, (0–0.09 s)), 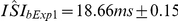, 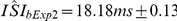) and significantly different for longer delays (140–240 ms, 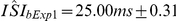, 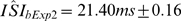, also see Table S2 in Text S1), with the fully diffused arrangement exhibiting larger 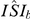 values than the fully clustered arrangement (Figure 5A). Intermediate delays (100–150 ms (0.1–0.15 s)) are characterized by a one-to-one mapping between the delay and the respective
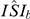
value for both arrangements. These findings suggest that the 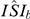 value (similar to the average *ff*) can be used to infer the synaptic arrangement (fully clustered or fully diffused) of signals that are separated by delays larger than 140 ms (0.14 s).

Results for the layer-specific clustering arrangements are also similar to the firing frequency findings: clustering in the SLM alone (Figure 5B, open circles) results in 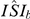 values which are more similar to the fully diffused case (Figure 5A, open circles) whereas clustering in the SR (Figure 5B, filled circles) results in 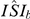 values which are more similar to the fully clustered arrangement (Figure 5A, filled circles. Also see Table S2 in Text S1). In all cases the average 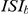 as a function of the delay can be well fitted by sigmoidal functions whose parameters are listed in Table 2. Taken together, these findings suggest that both spatial (arrangement) as well as temporal (delay between inputs) features of the layer-specific incoming signals are reflected in the average temporal properties (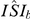) of the neuronal response. Moreover, assuming a rate code for information transfer, the average firing frequency and/or the average 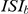 value may be used to signal differences in the spatial arrangement (fully diffused vs. fully clustered) of EC and CA3 inputs that arrive with a delay of 140–450 ms (0.14–0.45 s).

**Table 2:** Sigmoidal equations used to fit the average burst ISI curves in Figure 4.

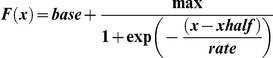	F_Exp_1_(x) ±s.d.	F_Exp_2_(x) ±s.d.	F_Exp_3_(x) ±s.d.	F_Exp_4_(x) ±s.d.
base	17.942±0.138	17.85±0.0853	17.169±0.119	18.502±0.104
max	6.7872±0.216	3.35±0.127	4.0077±0.166	6.2032±0.187
xhalf	131.51±2.1	125.03±2.44	116.03±2.32	142.06±2.09
rate	14.298±1.85	13.117±2.13	9.1361±2.01	18.96±1.88

#### The sequence of burst inter-spike-intervals

**Figure 6:**
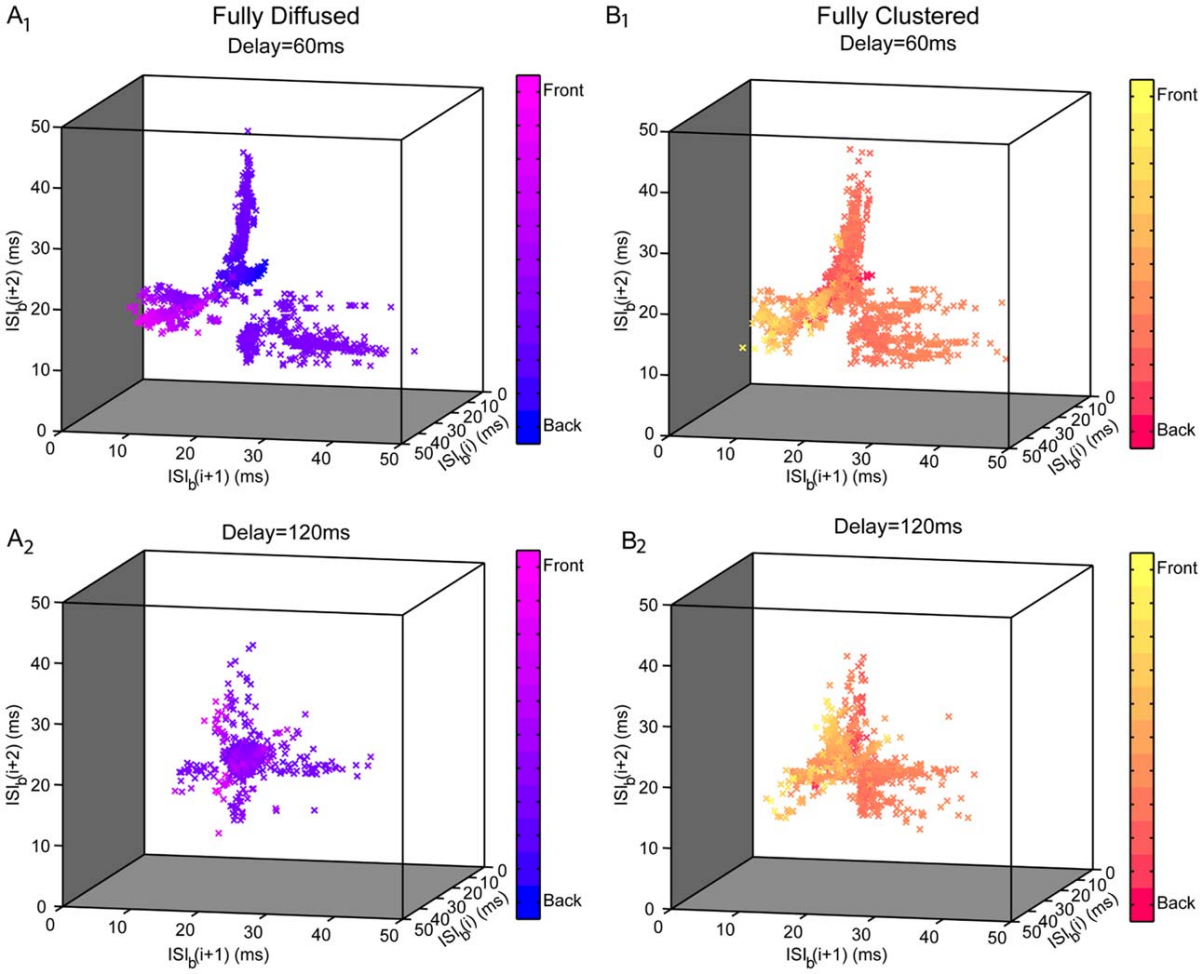
3-D return maps of successive 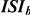 values. Return maps for fully diffused (A) and fully clustered (B) synaptic arrangements and two different delays (60 ms (0.06 s) top graphs and 120 ms (0.12 s) bottom graphs), across the 62 repetition trails (see Methods). Color has been added as a marker of depth. The number of points plotted in the different panels is A_1_: 2156, A_2_: 718, B_1_: 2230 and B_2_:1213. For a delay of 60 ms (0.06 s), model responses to diffused stimuli (A_1_) form a more structured map than responses to clustered synapses (B_1_), whereby the 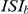 values are condensed in several tight groups. This grouping indicates a relationship between preceding and future spike occurrences for diffused signals and appears to fade in both arrangements when the temporal delay increases to 120 ms (0.12 s) (A_2_ and B_2_).

Although informative, neither the average firing frequency nor the average burst inter-spike-interval are sufficient to discriminate between fully clustered versus fully diffused signals that arrive within short delays. However, an analysis of the succession of inter-spike-intervals within each burst (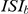) using three-dimensional return maps for two different delays (60 and 120 ms (0.06–0.12 s)) and two synaptic arrangements (fully diffused vs. fully clustered, see Methods) reveals that the successive 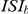 values could be used to encode differences in the spatial arrangement of incoming signals. As shown in Figure 6, the 3-D return maps of the model responses to fully diffused incoming signals form a much more structured map compared to clustered inputs (see Table 3 for a quantitative comparison). Furthermore, the respective maps for short delays reveal the formation of a few tight elongated groups which fade as the delay between SLM and SR signals increases (Figure 6 and Figure S1 in Text S1). This grouping of past, current and future 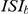 values for short delays indicates different degrees of variation in spike time occurrence as well as correlation between preceding, current and/or future action potentials. Since clustered arrangements are associated with higher entropy maps than the respective diffused cases (see Table 3), these findings suggest the existence of an underlying rule that characterizes the cell's firing pattern, which appears to be more prominent in fully diffused than fully clustered synaptic arrangements.

**Table 3:** Comparison of 3-D return maps for fully clustered and fully diffused arrangements.

	Diffused synapses		Clustered synapses
Delay (ms)	Normalized entropy	KS test	Normalized entropy
D = 20	0.498	p = 0.19	0.567
D = 60	0.473	p = 0.35	0.523
D = 100	0.537	p = 0.1	0.601
D = 120	0.476	p = 0.02	0.585
D = 160	0.414	p = 0.08	0.416

To quantify the difference between 3-D return maps we use the normalized entropy statistic [118] and the two-sample Kolmogorov-Smirnov test (KS test) between delay pairs. Entropy for clustered arrangements is larger compared to diffused arrangements indicating higher variability of the former responses. KS test is applied on the density histograms of each set.

**Figure 7:**
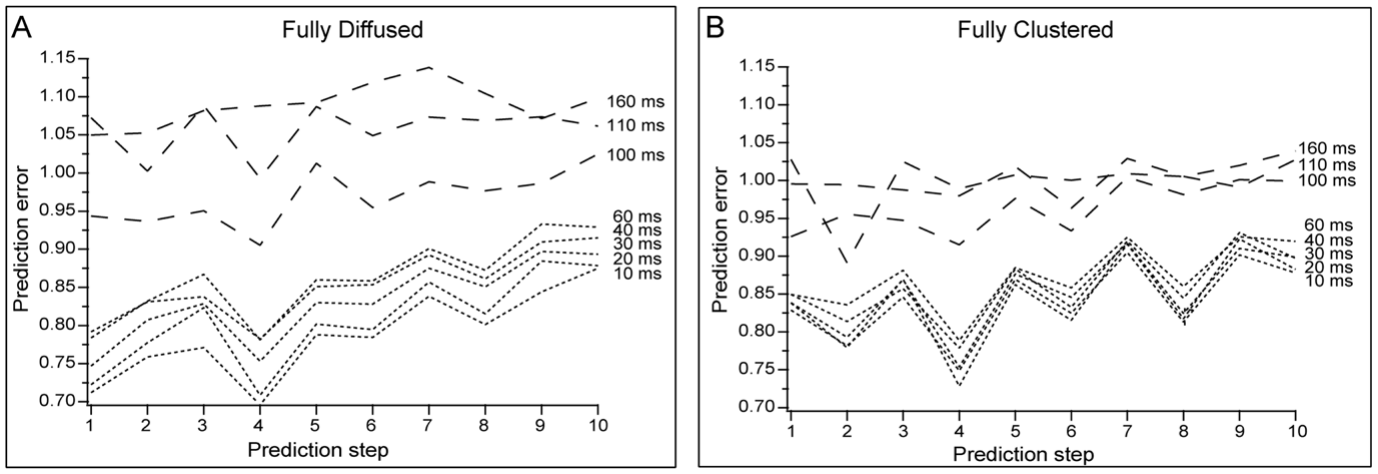
The sequence of 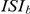 values contains predictive information about future spike occurrences. Nonlinear analysis of successive 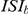 values for fully diffused (A) and fully clustered (B) synaptic arrangements and different delays. Each prediction step represents successive future 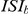 values, with step one corresponding to prediction of the next spike occurrence. For delays less than 100 ms (0.1 s), the prediction error for the first step is lower in the fully diffused (A) than the fully clustered arrangement (B), suggesting that the occurrence of the next spike can be estimated with higher accuracy in fully diffused than fully clustered arrangements. However, as the delay increases beyond 100 ms (0.1 s), the prediction error for both cases becomes close to/or larger than one, indicating that predictability is very poor. The predictability for future spike occurrences decreases for both arrangements along the prediction horizon.

To measure the degree to which future spike timings (estimated using successive 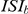 values) can be predicted based on the history of responses to similar inputs we next use non-linear time series analysis [57]. Specifically, for each delay (0–240 ms) and synaptic arrangement (fully clustered or fully diffused), we quantify prediction accuracy by grouping together all firing patterns (i.e. all 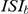 values) resulting from the 62 repetition trials (one pattern after the other) and estimating the prediction error (see Methods). As evident from Figure 7, the prediction error in short delays is smaller compared to long delays, and therefore, the predictability is better in short delays, where the two incoming signals overlap in time. Furthermore, the prediction error for diffused arrangements is slightly smaller compared to the clustered arrangement, suggesting that for delays shorter than 100 ms (0.1 s), at any given time, the occurrence of the next spike can be estimated with higher accuracy in fully diffused than fully clustered arrangements. This is in agreement with earlier findings [19], [20] showing that diffuse signals often fail to engage dendritic nonlinearities and are integrated in a linear averaging manner. This averaging over a larger number of synapses reduces the trial-to-trial variability in the somatic response, leading to better predictability. On the contrary, clustered signals lead to a higher incidence of local dendritic nonlinearities which are generated by a smaller number of synapses each, leading to greater variability in the somatic response and subsequently poorer predictability. To assess the extent to which somatic responses vary, we used the Local Variation (*LV*) metric [58] and compared all repetition trials for each synaptic arrangement and a given temporal delay (see section **Variability of individual neuronal responses** in Text S1).

Overall, for short delays, activation of synapses in a diffused manner results in responses which are very regular and similar to each other while activation of synapses in clusters induces highly variable firing patterns. Consequently, the sequence of inter-spike-intervals within bursts is likely to contain discriminatory information about the spatio-temporal features of incoming signals.

#### The sequence of burst inter-spike-intervals has discriminatory power

**Figure 8:**
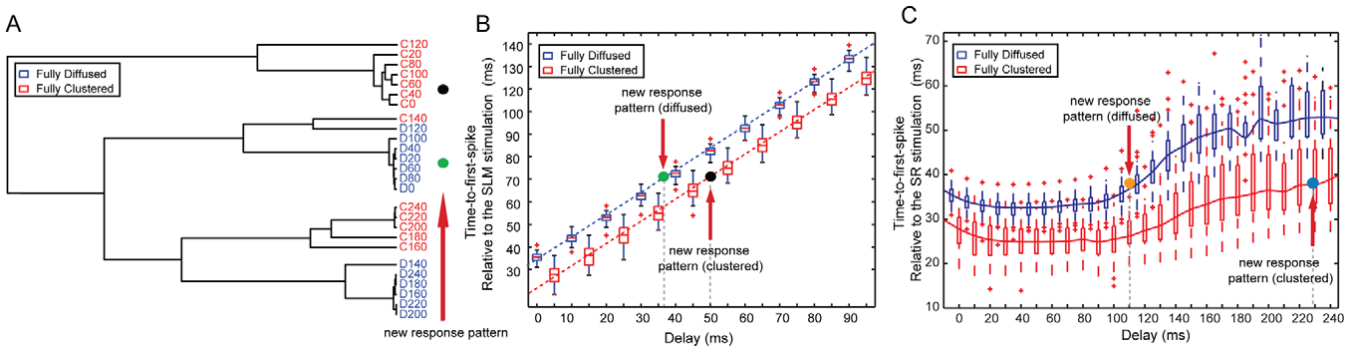
Model responses contain discriminatory information about the spatio-temporal characteristics of the input. A. Hierarchical clustering (complete linkage, Spearman Rank correlation) of median 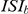 values for fully diffused and fully clustered arrangements and different temporal delays. With the exception of one case (fully clustered, delay = 140 ms (0.14 s)), responses to fully clustered inputs (red) are clearly separated from responses to fully diffused inputs (blue) for all delays. Moreover, responses for long delays (140–240 ms, (0.14–0.24 s)), are arranged in two sub-clusters each corresponding to the fully clustered and fully diffused case, respectively. Responses for short delays (0–120 ms, (0–0.12 s)) are clearly separated from responses for long delays and organized into distinct groups, one for each synaptic arrangement. Green and black dots represent two new response patterns which are found to be located within the fully diffused (green) and fully clustered (black) short delay sub-clusters. B. Time-to-first-spike box plot for fully diffused (blue) and fully clustered (red) stimuli. Horizontal red bars represent the medians for each case. For delays less than 100 ms, (0.1 s), occurrence of the first spike happens significantly faster in clustered than diffused stimuli. Given information about the spatial arrangement of two new response patterns (black: clustered, green: diffused), the delay between SLM and SR stimulation can be inferred using the time-to-first-spike lines when this is measured relative to the SLM stimulation, even if the time-to-first-spike value is identical for both responses. C. Same as B but for all temporal delays tested (0–240 ms) and with time-to-first-spike measured relative to the SR stimulation. In this case the modulation induced by the temporal delay is more subtle and non-linear. Discrimination of synaptic arrangement remains clear based on the time-to-first-spike values; however, prediction of the exact temporal delay from the time-to-first-spike values becomes hazy.

Our next goal was to investigate whether the information contained in the sequence of inter-spike-intervals within bursts is sufficient to discriminate between inputs that differ in their spatial and temporal characteristics. We address this question for the fully clustered and fully diffused synaptic arrangements and delays of 0–240 ms (0–0.24 s) by constructing a feature vector containing the successive 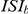 s along the 10-second response pattern of the model, averaged over the 62 repetition trials (see Methods). This procedure results in 13 different ‘signatures’ for each of the two synaptic arrangements, where each signature corresponds to a different delay. Using hierarchical clustering (complete linkage, Spearman Rank correlation), the 26 signatures are grouped to form the dendrogram shown in Figure 8A. With the exception of one case (fully clustered, delay of 140 ms (0.14 s)), responses to fully clustered inputs (red) are clearly separated from responses to fully diffused inputs (blue) for all delays. Specifically, the responses for delays between 140–240 ms (0.14–0.24 s) are arranged in two sub-clusters, each corresponding to the fully clustered and fully diffused case, respectively. Both of these sub-clusters are clearly separated from responses for delays between 0–120 ms (0–0.12 s), which are in turn organized into distinct groups, one for each synaptic arrangement. These findings show that the firing pattern of the model cell, and in particular the succession of inter-spike-intervals within bursts contains discriminatory information regarding the spatial arrangement (clustered or diffused) and the temporal characteristics (delay of 0–120 ms or 140–240 ms (0–0.12 s or 0.14–0.24 s)) of incoming signals. Therefore, the model neuron might use the sequence of 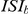 values of a given response pattern to infer whether the incoming stimuli activated synapses in a clustered or diffused manner and roughly estimate the delay that SLM activation preceded that of SR.

#### Time-to-first-spike

The time delay before the onset of activity following a stimulus (time-to-first-spike) is a feature often associated with temporal information coding in cortical neurons [59]. This delay is usually attributed to the dynamics of temporal and spatial summation of synaptic currents leading to the initiation of an action potential and has been suggested to convey behaviorally relevant information such as sound location [60] or the spatial structure of images [61]. The specific location of activated synaptic contacts throughout the dendritic tree has also been suggested to influence the time-to-first-spike (*ttfs*) value and perhaps serve as a mechanism for tagging inputs according to their respective delay in initiating somatic firing [62]. It is thus likely that changes in the spatial arrangement of incoming signals have a measurable effect on the *ttfs* value in the model cell, even in the absence of a difference in the average *ff* or 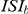 values. To address this question we compare the timing of the first spike under activation of the SR alone versus SR and SLM layers (simultaneously) and find that spiking occurs much earlier if both layers are activated (approximately 20 ms (0.02 s) earlier, data not shown). We then measure the *ttfs* value (with respect to SLM stimulation) for the fully clustered and fully diffused synaptic arrangements and delays between 0–100 ms (0–0.1 s). We focus on short delays because for this range both fully clustered and fully diffused signals generate bursting responses with nearly identical average *ff* (Figure 2A) and average 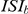 (Figure 5A) characteristics. We find that the *ttfs* value (taken over the 62 trials) is significantly smaller for clustered (Figure 8B, red) than diffused (Figure 8B, blue) signals for all delays between 0–100 ms (p<0.0001, t-test was performed for every *ttfs* pair corresponding to a single delay). This suggests that when synapses are activated in clusters, the cell responds much faster than when synapses were activated in random locations throughout the dendritic tree, while the average characteristics of the response signal (*ff* and 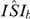) remain the same. This increase in response speed for clustered signals is likely to result from stronger dendritic activation which subsequently leads to faster and larger somatic depolarizations. As shown in previous work, clustered inputs engage dendritic nonlinearities (NMDA/calcium spikes) to a much larger extent than diffused signals [19], [20].

Moreover, when measured relative to the SLM stimulation (Figure 8B), the *ttfs* value for both arrangements seems to be linearly associated with all temporal delays up to 100 ms. Since this linearity is likely to reflect the delay between the two input streams, we also measured the *ttfs* relative to the SR stimulation (Figure 8C). We found that in the latter case, the *ttfs* values remain larger for clustered vs. diffused inputs for each delay, over all delays tested (0–240 ms (0–0.24 s)) but the modulation induced by the delay is more subtle and non-linear. We consider measuring the *ttfs* from the SLM stimulation, as opposed to the SR stimulation, more relevant to this analysis since layer V EC cells, which are the main targets of the CA1 output (together with the subiculum) [63], have no connections with the CA3 region and are thus not likely to have access to the information about SC activation. On the contrary, these cells have connections with layer III EC neurons [64], [65] (the ones projecting to the SLM layer) and are thus more likely to have information about the time of SLM input to the CA1. Our findings show that the *ttfs* contains information about the delay between layer III activation and the CA1 response, when the latter also contains SC input information.

The above findings suggest that for a given delay between 0–2.40 ms (0–0.24 s), a single feature of the neuronal response, i.e. the time-to-first-spike, is sufficient to infer the spatial arrangement of activated synapses. Furthermore, for any delay between 0–240 ms (0–0.24 s), the sequence of 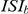 values can be used to infer the arrangement of incoming contacts by looking at the position of the new pattern on the dendrogram of Figure 8A (green or black dot). Once the arrangement has been inferred, the time-to-first-spike value can be used to approximately estimate the delay between SLM and SR activation by looking at its position on the respective line (green or black dot for delays less than 100 ms, orange or blue dot for delays larger than 100 ms) of Figure 8B (or Figure 8C). Therefore, given the specific stimulation protocol, a temporal code that consist of the sequence of 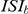 and the *ttfs* values carries sufficient information for inferring both the spatial (fully clustered versus fully diffused) as well as the temporal (approximate delay) characteristics of cortical and intra-hippocampal inputs.

**Figure 9:**
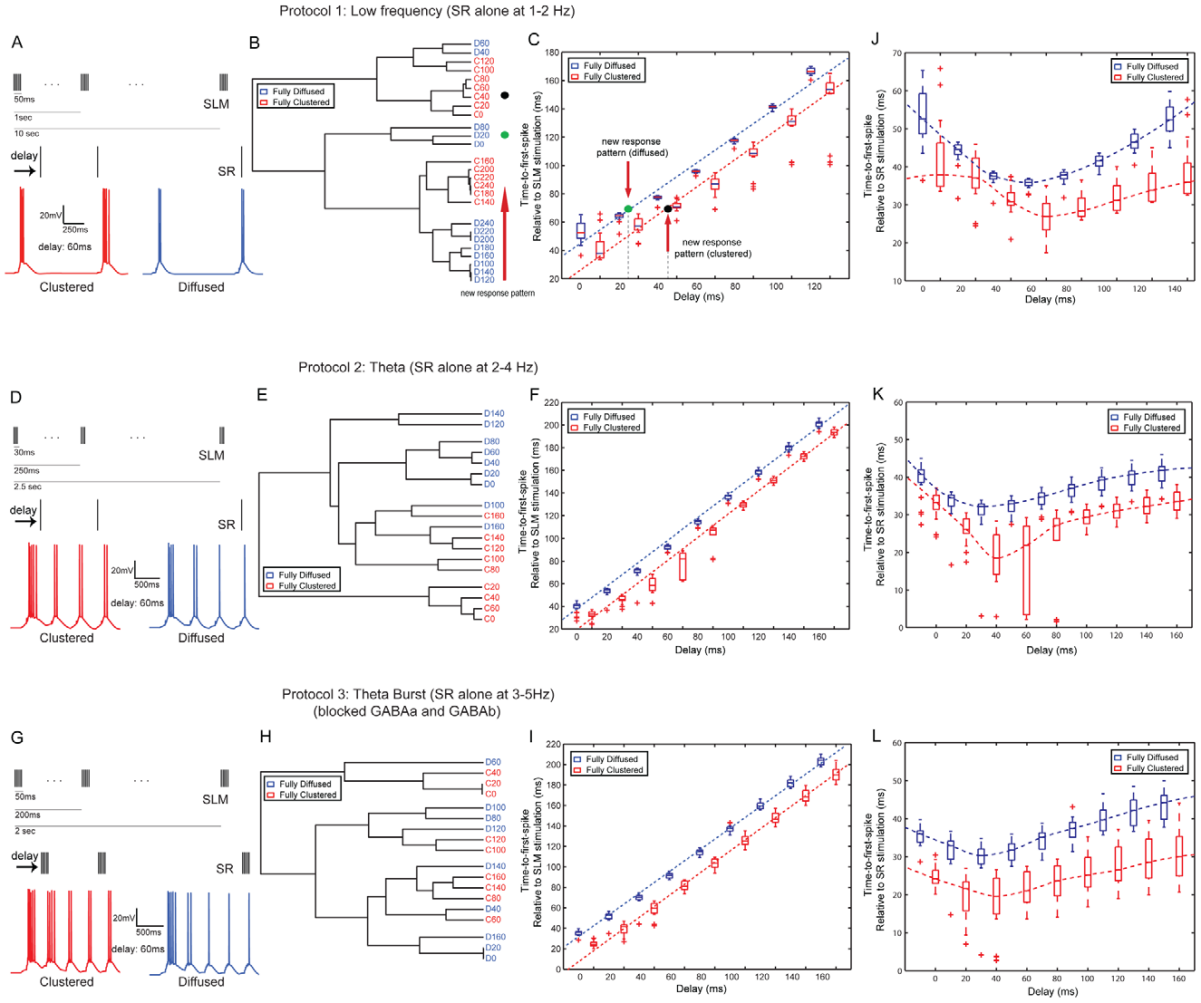
Robustness of spatio-temporal encoding across stimulation protocols. A, D, G. The three different stimulation protocols. B, E, H. Hierarchical clustering (complete linkage, Spearman Rank correlation) of median 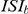 values for fully diffused and fully clustered arrangements and different temporal delays for each protocol. For Protocols 1 and 2, responses to fully clustered inputs (red) are clearly separated from responses to fully diffused inputs (blue) for all delays, with the exception of one-two cases. Discrimination is not as clear, however, for Protocol 3. As in Figure 8, green and black dots (in B) represent two new response patterns which are found to be located within the fully diffused (green) and fully clustered (black) short delay sub-clusters. C, F, I. Time-to-first-spike (relative to the SLM stimulation) box plots for fully diffused (blue) and fully clustered (red) stimuli for each protocol. Horizontal red bars represent the medians for each case. Occurrence of the first spike is significantly faster in clustered than diffused stimuli, for all three protocols. C. As in Figure 8, given information about the spatial arrangement of two new response patterns (black: clustered, green: diffused) the exact delays between SLM and SR stimulation can be inferred using the time-to-first-spike lines, even if the time-to-first-spike value is identical for both responses. J, K, L Same as C, F, I but with the time-to-first-spike measured relative to the SR stimulation. In this case, the temporal delay leads to a much more subtle, non-linear modulation of the time-to-first-spike values. Discrimination of synaptic arrangement remains clear but prediction of the temporal delay from the time-to-first-spike value becomes hazy.

It has been suggested that a small *ttfs* value may signal the presence of a strong input pattern [66], [67], [68], [69]. Our results are in agreement with these findings for signals leading to significantly different average responses. Specifically, stimuli with the same synaptic distribution and activation delays, but different synaptic strengths are associated with different *ttfs* values, with stronger stimuli (Protocol 3) leading to smaller *ttfs* (compare Figure 9, panels C, I). However, for stimuli that lead to similar average responses (Protocol 1, delay 0–100 ms, Figures 2A and 5A), the *ttfs* may be used to encode another characteristic of the input. According to our findings, when a rate code (average *ff* or 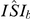) is not sufficiently informative, the time-to-first-spike contains discriminatory information about the synaptic distribution of incoming signals as well as the temporal latency separating the activation of the two layers.

#### Robustness of the spatio-temporal feature encoding across different stimulation protocols

**Figure 10:**
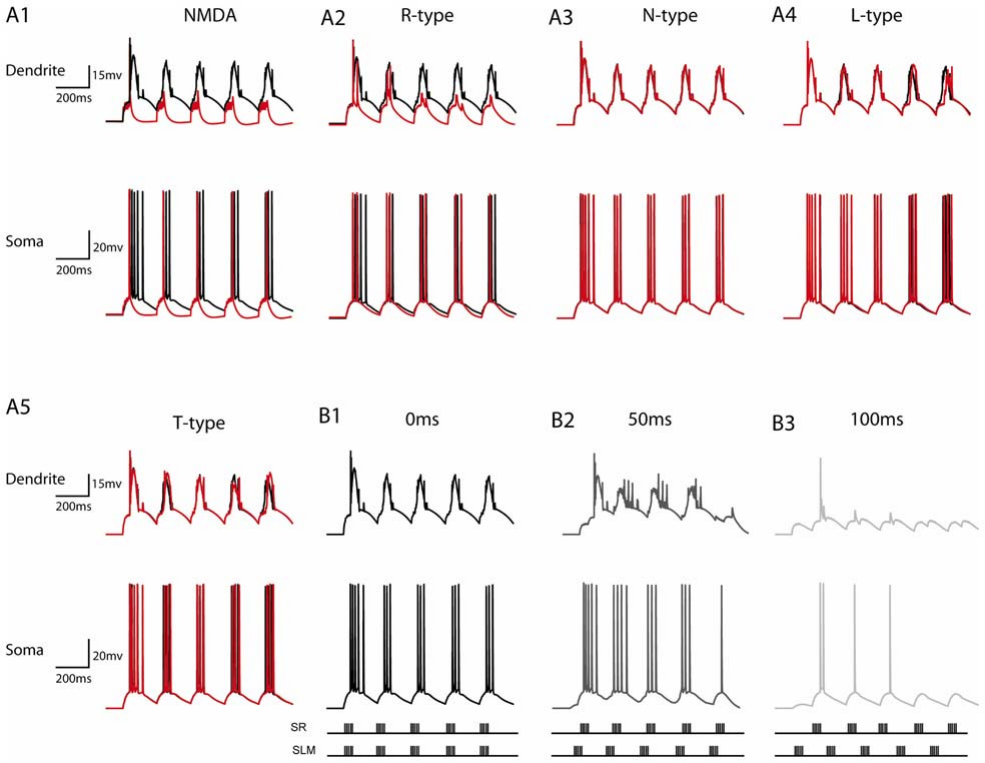
Mechanisms underlying somatic bursting. A. Representative traces from the model (somatic, dendritic at 300 microns) showing the effect of mechanism blockade (90% reduction in conductance) on the generation of dendritic plateau potentials and somatic bursts using Protocol 3. A1. Blockade of NMDA currents results in complete elimination of dendritic plateau potentials as well as somatic bursting. Note that AMPA currents were increased to counteract excitability reduction from the NMDA blockade when each of the pathways was stimulated individually, indicating that dendritic plateau potentials are NMDA dependent. A2. Blockade of R-type currents (caR and caRH) severely reduces the appearance and width of dendritic plateau potentials as well as somatic bursting. A3–A5. Blockade of N-type, T-Type and L-type currents has a negligible effect on dendritic and somatic responses, indicating that these mechanisms do not significantly contribute to dendritic plateau potentials or somatic bursting. B. Dependence of dendritic plateau potentials and somatic bursting on the temporal offset between SC and PP inputs. Traces showing simultaneous stimulation (0 ms, B1) produce large, long-lasting plateau potentials in the dendrites which diminish in size and duration as the two inputs are separated in time (by 50 ms, B2 and 100 ms, B3). Somatic bursting follows the same trend.

The above findings suggest that the time-to-first-spike and the sequence of 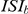 can be used to infer the spatial and temporal characteristics of the incoming stimuli. However, since these findings were based on a single protocol, this encoding ability of the neuron could be specific to the protocol characteristics (for example, delay of inhibitory input compared to excitatory input, frequency and number of events used for SLM/SR activation etc). To investigate this issue, we first slightly modify the inhibitory synapse connectivity and activation pattern of the model cell to account for (a) inhibitory contacts activated by SC inputs and terminating on the dendrites in the SLM layer (perforant path associated cells) [31] (b) inhibitory contacts activated by PP inputs and terminating on the soma (apical dendrite innervating cells) [34] and (c) a 2.5 ms (0.0025 s) delay between the activation of excitatory and inhibitory inputs [70] to account for the di-synaptic activation of PP→interneuron→CA1 and SC→interneuron→CA1 pathways. We then use different protocols for stimulation of the distal dendrites in the SLM, where we vary the number of events (3 or 5) during the 100Hz-burst as well as the frequency by which these bursts are repeated (1 Hz, 4 Hz and 5 Hz). Furthermore, we vary the protocol by which synapses in the SC layer are stimulated and we use either low frequency (single spikes at 1 Hz), theta cycle (single spikes at 4 Hz) or theta burst (100 Hz bursts repeated at 5 Hz), as detailed in the Methods. Note that the theta burst protocol is performed under conditions of reduced inhibition, as described in [41]. We find that all protocols induce somatic bursting for short delays but only the first protocol generates spike blocking when the delay between PP and SC signals is longer than 180 ms (0.18 s) (see Figure 10, and Figure S2 in Text S1), in accordance with our earlier results. Moreover, all three protocols have time-to-first-spike values in which model responses are initiated faster for clustered than diffused signals (see Figure 9C,F,I,J,K,L), as was the case with the initial protocol (see Figure 8B). In addition, the time-to-first-spike (relative to the PP input) is linearly associated with the SC signal delay and is statistically different between clustered and diffused signals corresponding to the same delay, for all protocols tested (including the initial shown in Figure 8B and Protocols1-3 shown in Figure 9C,F,I).

Importantly, the observed linearity in *ttfs* graphs suggests that model responses are likely to be driven by the SC signal, which arrives progressively later than the SLM input. To investigate this possibility, we also measured the *ttfs* relative to the SC signal and found a different, more subtle modulation of the *ttfs* parameter. Panels J-L in Figure 9 show the *ttfs* graphs evaluated relative to the SC input for each of the three protocols. In all cases, *ttfs* for diffused arrangements are significantly larger than respective values for clustered arrangements, indicating that the *ttfs* value may encode synaptic arrangement. However, for any given synaptic arrangement, the modulation of *ttfs* values by the temporal delay is much smaller (fitted curves are not monotonic), indicating that *ttfs* relative to the SC signal is not a reliable predictor of the temporal delay between SR and SLM activation. On the contrary, comparison of *ttfs* curves across protocols shows that for a given synaptic arrangement and activation delay, the *ttfs* value is smaller in Protocols 2 and 3 (strong input patterns) than Protocol 1 (weak input patterns) (p<0.0001, Protocol 1_ttfs_ vs. Protocol 2_ttfs_, for clustered and diffused synapses, delays  = 0, 20, 40, 60 ms, Mann-Whitney U-test), supporting earlier findings that this parameter also carries information about the intensity of the stimulus [68], [70], [71], [72].

Finally, discrimination of burst inter-spike-interval sequences between clustered and diffused signals is also maintained in Protocols 1 and 2, and becomes fuzzy in Protocol 3 (see dendrograms in Figure 9B, E, H). Note however that Protocols 1 and 2 are performed under control conditions, while Protocol 3 is performed under conditions of reduced inhibition. Even in this case, the time-to-first-spike is clearly different between clustered and diffused signals (irrespectively of the way it is measured). The discrimination of different arrangements and delays via the use of 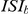 sequences, however, becomes more difficult, highlighting the importance of inhibitory input for the reliable encoding and transmission of input patterns that differ in their spatio-temporal characteristics. Taken together, these findings show that the encoding capabilities of our model cell with respect to spatio-temporal characteristics of incoming stimuli remain sound across four different stimulation protocols.

#### Model validation regarding the mechanisms underlying bursting for Protocol 3

According to our earlier predictions [40] which are in line with the recent experimental findings of Takahashi and Magee [41], somatic bursting is evident when PP and SC inputs are temporary close due to the generation of dendritic plateau potentials. Moreover, the experimental data show that the biophysical mechanisms underlying the generation of these plateau potentials seem to be the NMDA and R-type calcium currents, while none of the T-type, N-type or L-type currents appears to contribute significantly. To test the validity of our model with respect to the experimental findings of Takahashi and Magee, 2009, we used the same stimulation protocol (Protocol 3) and recorded the somatic as well as dendritic responses when all of the above mechanisms were blocked by 90%. Figure 10 shows representative traces form the model under the influence of the various blockers. As evident from the figure, blockade of T-type, N-type and L-type currents does not affect model responses while blockade of NMDA and R-type calcium currents eliminates dendritic plateau potentials as well as somatic bursting.

The effect of temporal dispersion between PP and SC inputs in the model was also investigated. As shown in Figure 10B, simultaneous activation of both pathways (0 ms, B1) produces large, long-lasting plateau potentials in the dendrites of the model cell which diminish in size and duration as the two inputs are separated in time (by 50 ms, B2 and 100 ms, B3). Somatic bursting follows the same trend. All of the above findings are in close agreement with the results of Takahashi and Magee [41], under the same stimulus conditions. One difference between our findings and their findings is that our model does not incorporate plastic synapses and thus it cannot reproduce the LTP induction associated with plateau potential generation. This plasticity phenomenon may also be responsible for the experimentally observed increase in dendritic plateau potential magnitude and duration within the stimulation protocol, after the first 1–2 bursts.

## Discussion

We have investigated the integrative properties of a detailed CA1 pyramidal neuron model under delayed and layer-specific synaptic stimulation, considering the spatial arrangement of activated synaptic contacts. We found that:

1. The model's firing pattern ranges from suppression to enhancement of baseline responses, depending on the spatio-temporal characteristics of incoming signals.
2. The delay between EC and CA3 inputs may act as a mechanism for alternating between excitability states: short delays lead to strong bursting while long delays lead to reduced firing.
3. For low frequency stimulation, synaptic arrangement has a layer-specific effect: clustering in the SLM promotes excitability reduction (spike blocking) while clustering in the SR promotes excitability enhancement (bursting).
4. Some spatio-temporal information of incoming stimuli is captured by a rate code (average 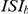 and *ff* of model responses).
5. Predictive information about the occurrence of the next spike is contained in the sequence of 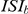 values and is more accurate for diffused than clustered signals.
6. Discriminatory information about the spatio-temporal characteristics of the input is contained in the intra-burst activity (sequence of 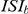 values) and the onset delay (time-to-first-spike) of the model's response across different stimulation protocols.
7. The time-to-first-spike contains discriminatory information about both the synaptic arrangement and the stimulus strength. When measured relative to the PP activation, it also contains discriminatory information about the activation delay between PP and SC pathways; when measured relative to the SC activation is shows a more subtle modulation by the abovementioned delay.

These findings suggest that a single CA1 pyramidal neuron may be capable of detecting and propagating several characteristics of the ongoing network activity via the modulation of its firing pattern, presumably reflecting the use of a temporal code for information transfer.

### Spike blocking: Understanding inhibition in the CA1 pyramidal neuron model

The soma-dendritic axis of CA1 pyramidal neurons is innervated by a plethora of GABAergic interneurons [73] whose activity influences local dendritic signals, thus promoting the functional compartmentalization of the dendritic arbor [74], [75] which in turn enhances the computational capabilities of individual pyramidal neurons [19], [20], [76], [77]. Together with the cooperation of the two major glutamatergic inputs in space and time, the single neuron model captures anatomical and functional characteristics of the CA1 microcircuit [78], [79]. In our model, inhibition plays a key role in the emergence of spike blocking. In line with experimental findings [12], this blockade of excitability depends on the strong, long-lasting, activation of GABA_B_-mediated currents originating at the SLM layer, presumably due to the EC-mediated activation of GABAergic neurogliaform interneurons [32]. An alternative explanation for the GABA_B_-mediated reduction in excitability has been found in neocortical pyramidal neurons where the elimination of somatic bursts resulted from the GABA_B_-induced blockade of dendritic calcium spikes [15]. In accordance with these findings, elimination of dendritic calcium spikes in the model cell (shown in Figure 10) greatly impairs burst activity at the cell body. It is likely that this pathway-specific inhibitory network acts as a gate that preferentially isolates certain cortical inputs from delayed intra-hippocampal signals thus preventing their association [43].

### Synaptic arrangement and information coding

An important aspect of this work concerns the role of synaptic arrangement in shaping the firing pattern of the model neuron. Clustered activation of synapses in particular, seems to play an important role in information processing in our model. There is now cumulating evidence suggesting that synaptic clustering not only facilitates neural computations [80] and information processing [81] but it can emerge as a result of learning and memory processes [82]. Moreover, lamina-specific axonal branching in thalamocortical connections and remodeling processes can be regulated by neural activity [83]. As recently shown by Le Bé and colleagues, there is extensive rewiring in the neonatal neocortex which includes the spontaneous as well as evoked formation of new, fully functional, synaptic contacts within clusters of interconnected pyramidal neurons [84]. The authors also showed that following stimulation, weaker connections are selectively eliminated suggesting that this form of plasticity enables the evolution of the microcircuit connectivity by natural selection as a function of experience. Finally, according to the clustered plasticity model of Govindarajan et al., when synapses are activated in clusters within a branch they facilitate the establishment of long term memory engrams via the association of neighboring synapses that are strengthened or weakened through a tractable molecular process heavily dependent on local translational enhancement [21].

We find that in addition to the temporal interplay between the two input streams, the spatial distribution of activated synapses also impacts dendritic integration and neuronal output. This is in accordance with previous modeling and experimental studies where individual dendrites of pyramidal neurons were shown to combine incoming signals almost independently, in highly non-linear ways [16], [18], [19], [20], [85], [86], [87]. The significance of this compartmentalization in multiple, quasi-independent non-linear subunits is that it allows a single neuron to act as a two (or multi)-layer neural network, where information is first processed locally in the dendrites before reaching the somatic thresholding non-linearity, thus massively expanding its information processing capacity [20], [59]. While highlighting the powerful computations of dendrites and single neurons, the above studies did not examine whether these non-linear properties may also be used to determine the information content of neuronal output patterns as a function of realistic incoming signals. Here, we show that the firing pattern (sequence of 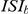 values) of a single pyramidal neuron model contains enough information to discriminate between realistic incoming signals whose spatial distribution is clustered within a few dendrites or diffused throughout the receiving dendritic layers (Figures 8A, 9B,E,H). This discrimination is possible even when the average firing properties (*ff* and/or 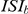) of neuronal responses are indistinguishable, indicating that a rate code may be insufficient for signaling subtle differences in the spatial organization of layer-specific stimuli. In addition to spatial discrimination, the firing pattern of the model cell can be used to estimate the temporal difference (0–120 ms vs. 140–240 ms, (0–0.12 vs 0.14–0.24 s)) between incoming signals impinging onto the SR and SLM dendritic regions (Figure 8A), thus conveying both temporal and spatial information of network inputs to the downstream cells. Importantly for delays smaller than 100 ms where a rate code fails completely, once the spatial arrangement of the inputs is inferred (Figures 8A, 9B,E,H) the latency until the initiation of a response (time-to-first-spike) relative to the SLM stimulation can be used to estimate the delay between the two layer-specific signals (Figure 8B, 9C,F,I). This is particularly important as it suggests that a single parameter of the CA1 pyramidal neuron output, i.e. the first spike latency, can be used to propagate to the cortex the temporal dispersion between the CA3 and EC network activities.

Assuming that recipient cells can decipher this propagated information, what could be the functional relevance of discriminating between clustered and diffused signals? Our working hypothesis is that each clustered activation of synapses signals the existence of a fingerprint representing a consolidated memory [21]. Activation of these fingerprints in the model amounts to re-activation (e.g. for retrieval/comparison purposes) of these memories. This hypothesis further implies that each clustered input to the model neuron is associated with a discrete mnemonic information tag. Activation of randomly distributed synapses on the other hand is assumed to represent a novel or non familiar information item which is not contained in the memory reservoir of the model cell. Under these assumptions, the information content of neuronal responses to clustered signals -measured by their variance [88]- should be higher than the one corresponding to diffused inputs. Our results show that diffused signals are associated with similar firing patterns (compact clouds in Figure 6A_1_, A_2_) and high predictability (low prediction error in Figure 7A), indicating that their information content might be relatively low. Clustered signals on the other hand generate more distinct neuronal outputs (large clouds in Figure 6B_1_, B_2_), thus hampering prediction attempts (higher prediction error in Figure 7B). These findings suggest that clustered signals are likely to carry more specific information than the ones activating random sets of synapses.

The fact that model responses to clustered signals systematically occur a few (∼10–15) milliseconds earlier than for diffused signals, is also in line with our working hypothesis. Direction-selective activity in monkey prefrontal cortex has been shown to occur earlier as a consequence of experience [89], suggesting that reactivation of existing memories (presumably in the form of clustered signals) leads to faster responses. Smaller latency in the onset of neuronal responses after experience induced paradigms has also been observed at the supplementary eye field of monkeys, in experiments exploring the processes of action selection [90]. Therefore, the smaller latencies found in model responses to clustered signals may indicate the presence of a mechanism for expressing anticipatory behavior.

### Single neuron information processing and neural codes

Our findings regarding the role of successive spikes within bursts in information transfer are in agreement with several other studies. In sensory systems, bursts not only facilitate synaptic transmission at unreliable synapses but they also enhance the transmission of sensory signals *in vivo* by carrying stimulus-specific information (for a review see [71]). For example, Oswald et al., report that burst ISIs encode stimulus amplitude,[72] in the electrosensory lobe of the weakly electric fish. Moreover, in primate primary visual cortex, different visual stimuli (contrast-related information) are associated with different classes of ISIs, with respect to their duration [68], [91]. High frequency bursts are also known to occur in the hippocampus and the CA1 region [92], [93] and they have been associated with information coding. Bursting in pyramidal place cells for example has been suggested to represent the location of the animal in space, both through the average firing rate [94] as well as the timing of bursts with respect to the theta cycle [95]. Here we show that both rate and temporal codes may also be used to compactly transfer such information to recipient cells in the cortex.

Numerous studies have shown that complex, behaviorally relevant operations can be performed by individual neurons [96]. Examples include orientation selectivity [97], [98], velocity tuning [96], [99], motion detection [100] and many more. These operations are realized via a set of rules or neural codes whereby the output signal of an individual neuron encapsulates and transmits information contained either in the average properties (average *ff*/*ISI*) or the precise temporal characteristics of its firing pattern [101], [102], [103]. We find that both strategies can be utilized by the model, but the latter conveys more information than the former. We show that a rate code (average *ff* and/or 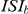) can be used to infer the spatial arrangement of inputs only if the SR signal is sufficiently delayed (120–160 ms, (0.12–0.16 s)). For smaller delays, signals that vary solely in their spatial arrangement are considered iso-response stimuli [104] and cannot be distinguished by a rate decoder. However, temporal information like the succession of burst inter-spike-intervals and the response onset latency, can be used to distinguish the spatial arrangement (clustered vs. diffused) of input signals across different stimulation protocols over a wide range of delays (0–240 ms, (0–0.24 s)). Thus, depending on the delay between layer-specific inputs both a rate and/or a temporal code may be successful in propagating spatio-temporal information to downstream cells.

### Limitations

Our detailed model reproduces closely the electrophysiological activity of CA1 pyramidal neurons. Nonetheless, sources of inaccuracy may have been introduced since the experimental data used to constrain the model are products of *in vitro* preparations. In that sense however, model limitations do not significantly differ from those of the *in vitro* preparations whose findings are readily replicated by the model. Moreover, the validity of previous model predictions has recently been established experimentally [16], [18], lending further support to its realism. Simplifications that have been adopted in this work include (i) a strictly postsynaptic phenomenological model of the GABA_B_ receptor desensitization [105] and (ii) uniform kinetic properties of same-type synaptic receptors throughout the model cell. Finally, the colocalization of axodendritic contacts (synaptic clustering) is assumed to result from an adaptive, learning-driven, mechanism. While this hypothesis was recently verified experimentally in the auditory system of barn owls [25] and previously in the hippocampal mossy fiber system of rats [106] [82] (see for a review [107]), it remains unclear whether such changes also occur in CA1 pyramidal neurons.

### Concluding remarks

We previously showed that the average firing rate of our model in response to high frequency Poisson trains can be predicted by knowing the number and dendritic location of activated synapses, via the use of a simple mathematical equation describing a conventional 2-layer neural network [20]. Here, we show that for different stimulation protocols activating synapses in the SLM and SR layers of the model cell, this prediction can be inversed: given the output pattern of the model, we can infer the arrangement of activated synapses as well as the temporal difference between activation of the two pathways. These findings suggest that a single CA1 pyramidal neuron may be capable of encoding and transmitting spatiotemporal information about the activity of the EC-hippocampal network to higher brain regions via the selective use of a rate or a temporal code. Whether this information-rich pattern can be decoded by recipient neurons in the subiculum and the deep layers of Entorhinal Cortex remains unclear, although both of these regions seem to be associated with the processing of complex input signals [108], [109], [110], [111]. The importance of our findings will become greater in light of pending experimental evidence such as *in vivo* estimates of the arrival delay between EC and CA3 signals at the CA1 region during learning and memory tasks and solid evidence supporting a direct role of clustered versus diffused activation of synapses in memory processes. However, even in the absence of these data, our modelling work sheds new light on how key features of pathway specific incoming signals can be propagated across neural networks: by showing that a single CA1 pyramidal neuron may act as a complex computational kernel where inputs are transformed -both in the time and frequency domains- in order to ensure their reliable and identifiable transmission.

## Methods

### Basic properties of the CA1 model

The compartmental model of the CA1 pyramidal neuron was implemented and run within the NEURON simulation environment [112]. The model is a refinement of a previously published model [19] and it contains a large number of ionic and synaptic mechanisms known to be present in these cells; specifically 15 different types of ionic currents and 4 different synaptic mechanisms (AMPA, NMDA, GABA_A_ and GABA_B_). Densities and distributions of the mechanisms included in the model are based on published empirical data and are fully described in Text S1. To replicate the prominent role of the GABA_B_ receptor in the spike blocking phenomenon, this mechanism has been modified to exhibit desensitization, a short-term type of plasticity, in response to high frequency stimulation (see Text S1).

### Layer-specific distribution of synaptic inputs

Synaptic inputs to the model cell were positioned within any or both of the two receiving layers corresponding to the Stratum Radiatum (SR) and the Stratum Lacunosum Moleculare (SLM) regions. The SR layer is defined as the apical trunk sections located within 13.40 and 292.06 microns (1.34·10^−5^ and 2.92·10^−4^ m) from the cell body and the apical oblique dendrites located within 0 and 300.94 microns (0 and 3.01·10^−4^ m) from the soma. The SLM layer is defined as the apical trunk sections located within 324.53 and 346.53 microns (3.25·10^−4^-3.47·10^−4^ m) from the soma and the apical oblique dendrites located beyond 419 microns (4.19·10^−4^ m) from the soma. In all cases location is estimated by measuring the perpendicular distance of the start point of each dendritic section from the soma.

The ratio of excitatory to inhibitory synapses is different between the two layers, according to the anatomical data of [30]: 
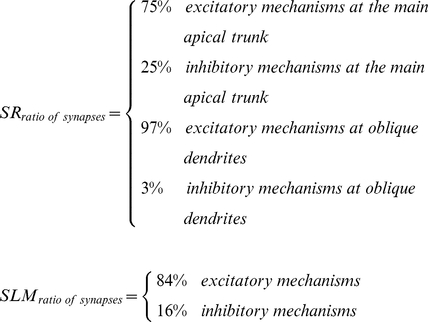



Each excitatory synapse consists of one AMPA and one NMDA receptor (co-localized) while each inhibitory synapse contains one GABA_A_ or one GABA_B_ receptor. Both types of inhibitory synapses are placed in the same dendrites. A fixed number of synapses were used in all simulations and they were distributed as detailed in Table 4.

**Table 4:** Distribution of synaptic mechanisms in the model cell.

	NMDA	AMPA	GABA_A_	GABA_B_
	receptors	receptors	receptors	receptors
SR main apical trunk	48	48	16	16
SR oblique dendrites	62	62	2	2
SLM	27	27	5	5
Soma	0	0	5	5

The SR main apical trunk section consists of the apical trunk dendrites located beyond 13.40 (1.34·10^−5^ m) and up to 292.06 (2.9206·10^−4^ m) microns from the soma whereas SR oblique dendrites correspond to the side branches found within 0 to 300.94 microns (3.0094·10^−4^ m) from the soma. The SLM layer contains the thick apical dendrites located within 386 to 424.75 microns (3.86·10^−4^ to 4.2475·10^−4^ m) from the soma and the side branches located beyond 419 microns (4.19·10^−4^ m) from the soma. The soma receives only inhibitory input.

### Synaptic currents

To account for the experimentally reported regional differences in synaptic currents the ratios of GABA_A_/GABA_B_ and NMDA/AMPA currents are gradually increased with growing distance from the soma. According to [113] the ratio of NMDA/AMPA EPSCs seems to increase with distance from the soma, reaching an almost two fold increase at SLM synapses as compared to SR synapses. In our model this increase is implemented as shown by Equations 1 and 2.
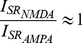
(1)

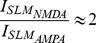
(2)


Inhibitory synaptic currents have also been shown to change with distance from the soma. According to [114] the 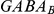-mediated current is significantly larger at the distal dendrites (located beyond 250 microns from the soma) compared to proximal ones whereas the same current is shunted by the 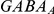 mediated current in the SR layer. This data is included in the model according to the Equations 3 and 4: 
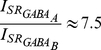
(3)

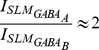
(4)


### Spatial arrangement of synaptic inputs within the SR and SLM layers

To study the effect of synaptic arrangement on the firing properties of the model neuron, we use four different synaptic arrangements. In the **fully diffused** arrangement (Exp 1), the location of each synapse is randomly selected among all dendrites of each layer and all possible positions along each dendrite. In the **fully clustered** arrangement (Exp 2), synapses are divided in equal-sized groups and positioned (clustered) within a few randomly selected branches (eight branches in the SR and four branches in the SLM). In the **SR clustered** arrangement, synapses within the SR layer alone are distributed in clusters whereas synapses in the SLM are randomly scattered (Exp 3). Finally, in the **SLM clustered** arrangement, synapses in the SLM layer alone are distributed in clusters whereas synapses in the SR are randomly scattered (Exp 4). In all cases, both the dendrites and the synapse positions within each dendrite were selected using a uniform distribution. Note that clustering refers to the positioning of synapses within a single dendrite and not tight grouping of contacts within a certain radius for each dendrite.

### Initial stimulation protocol

The initial stimulation protocol used in this work was introduced by [12]. Synapses in the SR layer are activated simultaneously at a frequency of 1 Hz, for a period of 10 s. Synaptic currents in this layer maintain the ratios described before and are calibrated to evoke a single supra-threshold event (action potential) for each input signal using a fully diffused synaptic arrangement. Due to variations in the synaptic arrangement between runs, the abovementioned stimulation of the SR layer may produce 2–3 action potentials. Synapses in the SLM layer are stimulated simultaneously with high frequency sub-threshold bursts (10 pulses at 100 Hz). Each burst is delivered at a frequency of 1 Hz for 10 s, similar to the SR stimulation (see Figure 1A, B). The somatic response to combined SR and SLM stimulation contains an initial excitatory phase and a long lasting inhibitory phase similar to that described by [12]. The activation of the two pathways is separated by a temporal delay ranging between 0–450 ms (0–0.45 s), where synaptic stimulation at the SLM layer always precedes SR stimulation.

### Data generation and analysis

Each different synaptic arrangement experiment (Exp. 1–4) was repeated 100 times, for each of the 34 different temporal delays (0–300 ms, with a 10 ms step, 350, 400, and 450 ms (0–0.3 s with a 0.01 s step, 0.35, 0.4 and 0.45 s)). From the pool of the 13600 recordings, we select those recordings where activation of SLM synapses alone results in sub-threshold events and activation of SR synapses alone results in action potential generation at a frequency of 1–3 Hz, both assessed at the soma. This filtering step resulted in a total of 62 recordings for each delay and each arrangement experiment.

The ***average firing frequency*** of the model cell is calculated according to the formula:
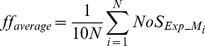
(5)and the ***average Inter-Spike Interval*** (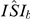) within bursts according to the equation:
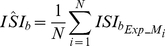
(6)where N is the number of trials (N = 62), *NoS* is the number of spikes counted over each trial, and Exp_M, M = 1,2,3,4 corresponds to the four different synaptic distribution experiments.

### Blockade of biophysical mechanisms

To evaluate the contribution of specific biophysical mechanisms in spike blocking and burst generation in the model neuron, we reduce their conductance by 90%. In experiments where the NMDA or GABA_B_ conductances are blocked, the AMPA/GABA_A_ conductances are enhanced in order to counteract the reduced excitation and inhibition, respectively.

### Model refinement and Robustness Analysis

To test the validity of our conclusions under different stimulus conditions, we used three additional stimulation protocols in a refined version of the model. The refinements included (a) the addition of inhibitory contacts activated by SC inputs and terminating on the dendrites in the SLM layer (10% of the inhibitory inputs to the SLM), (b) the addition of inhibitory contacts activated by PP inputs and terminating on the soma (10% of the inhibitory inputs to the soma), (c) a 2.5 ms delay between the activation of excitatory and inhibitory inputs, to account for the di-synaptic activation of PP→interneuron→CA1 and SC→interneuron→CA1 pathways.

#### Protocol 1 (low frequency)

SR is stimulated with 10 suprathershold pulses at 1 Hz, leading to a firing frequency of 1-2 Hz. SLM is stimulated with 10 events at 1 Hz, each event consisting of 5 subthreshold pulses at 100 Hz. The two layers are stimulated with a delay ranging from 0–350 ms (0–0.35 s), with the SLM activated prior to the SR. When activated together, the average firing frequency of the model ranges from 4–5 Hz to less than 1 Hz, depending on the delay and the synaptic arrangement as shown in Figure S2A in Text S1.

#### Protocol 2 (theta)

SR is stimulated with 10 suprathershold pulses at 4 Hz, leading to a firing frequency of 2–4 Hz. SLM is stimulated with 10 events at 4 Hz, each event consisting of 3 subthreshold pulses at 100 Hz. The two layers are stimulated with a delay raging from 0–160 ms (0–0.16 s), with the SLM activated prior to the SR. When activated together, the average firing frequency of the model ranges from 9–11 Hz to less than 2 Hz, depending on the delay and the synaptic arrangement as shown in Figure S2B in Text S1.

#### Protocol 3 (theta burst)

This protocol is used in [41] and was implemented in the model for validation purposes. SR is stimulated with 10 suprathershold pulses at 5 Hz, leading to a firing frequency of 3–5 Hz. SLM is stimulated with 10 events at 5 Hz, each event consisting of 5 subthreshold pulses at 100 Hz. The two layers are stimulated with a delay raging from 0–160 ms (0–0.16 s), with the SLM activated prior to the SR. When activated together the average firing frequency of the model ranges from 10–12 Hz to less than 5 Hz, depending on the delay and the synaptic arrangement as shown in Figure S2C in Text S1. As in [41], both GABA_A_ (decreased by 70%) and GABA_B_ (decreased by 80%) conductances are reduced in this protocol.

### Return Maps

To investigate the existence of inter-relationships between successive 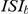 values, we produce three-dimensional Return Maps for the fully clustered and the fully diffused synaptic arrangements and five different delays (20, 60, 100, 120 and 160 ms (0.02, 0.06, 0.1, 0.12, 0.16 s). This is done by constructing separate vectors each of which contains the series of 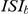 values for a specific delay and a specific arrangement over the 62 repetition trials:
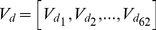
(7)where 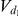 is the series of successive 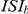 values obtained from the first trial, 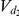 is the series of 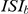 values obtained from the second trial and so on. Each vector is then used to generate a 3D graph showing each 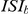, as a function of its preceding and succeeding 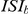 values (Figure 6 and Figure S1 in Text S1). Regions in this 3D space where data points appear in tight clusters suggest high similarity and/or correlation between successive 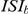 values. To quantify variability and differences between the return maps we used the normalized entropy statistic [115] and the two-sample Kolmogorov-Smirnov test for evaluating significant differences between density histograms of the *V_d_* vectors.

**Hierarchical clustering** of 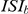 patterns for fully clustered and fully diffused arrangements and in all Protocols was performed using the dendrogram function in Matlab (Similarity function: Spearman Rank correlation, Linkage: Complete). For each arrangement and delay, the median 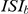 pattern is estimated by taking the median of each 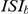 value throughout the somatic firing response over the repetition trials.

**Time-to-first-spike** for fully clustered and fully diffused arrangements as a function of the delay was assessed for all Protocols using the boxplot function in Matlab. Boxes indicate the lower quartile, median and upper quartile values while the lines extending from each end of the boxes (whiskers) show the extent of the rest of the data. Outliers are data with values beyond the ends of the whiskers. If there is no data outside the whisker, a dot is placed at the bottom whisker.

**Variability of individual neuronal responses** was done to investigate the variability of responses within the same synaptic arrangement using the local variance metric described in [58].

**Prediction error** analysis was done to investigate the presence of deterministic structure in the data using the non-linear prediction algorithm by Kantz and Schreiber [116]. Analysis of the 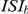 time series using this method (TISEAN software) is based on the theory of non-linear deterministic dynamical systems. The parameters used were: *time delay*  = 6 (calculated from the autocorrelation function), *embedding dimension*  = 10 (calculated with the false nearest neighbors from TISEAN software), *number of neighbors*  = 8, *prediction step*, 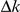  = 10. The prediction error is calculated as the average value of the squared difference between the predicted 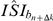 and the real 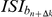 future values [54]:
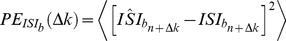
(8)

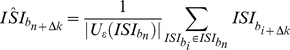
(9)where 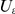 denotes the adjacent neighbor in which at least 8 neighbors are within its limits.

### Simulation methods

All simulations in this study were carried out within the NEURON simulation environment [112], using the variable time step method (CVODE). Simulations were performed using two xeon servers and a cluster of 64 dual Opteron 242 with 1 Gbyte main memory CPU systems interconnected with a Gigabit Ethernet.

## Supporting Information

Text S1 This file contains tables and figures (both with titles and legends) than have been mentioned in the main text. That is, Tables S1–S5, Figures S1–S6. It also describes in detail some analysis that was not included in the article and the mathematical formulas of the mechanisms used in the model (ionic currents, receptors, passive properties) as well as their distribution throughout the model neuron.
(6.97 MB DOC)
