# Supplementary material for: Encoding of Spatio-Temporal Input Characteristics by a CA1 Pyramidal Neuron Model
Source: PLoS Comput Biol. 2010 Dec 16;6(12):e1001038. doi: 10.1371/journal.pcbi.1001038 (PMC3002985; doi:10.1371/journal.pcbi.1001038)
Supplement: Text S1 — This file contains tables and figures (both with titles and legends) than have been mentioned in the main text. That is, Tables S1–S5, Figures S1–S6. It also describes in detail some analysis that was not included in the article and the mathematical formulas of the mechanisms used in the model (ionic currents, receptors, passive properties) as well as their distribution throughout the model neuron. (6.97 MB DOC) [file pcbi.1001038.s001.doc]

**Text S1**

## Statistical Analysis of average firing frequency (*ff*) and burst inter-spike-interval (*ISI_b_*) curves

**Table S1.** Anova tests for the average firing frequency curves shown in Figure 2.

In cases where the curves did not differ significantly, the average p value and the respective standard deviation is reported.

| **Exp pair and delays** | **0-90 ms** | **100-190 ms** | **200-300 ms** |
| --- | --- | --- | --- |
| Exp1-Exp2 | p=0.518 ± 0.160 | p<0.001 | p<0.001 |
| Exp3-Exp4 | p<0.001 | p<0.001 | p<0.001 |
| Exp2-Exp3 | p=0.335 ± 0.166 | p=0.375 ± 0.245 | p=0.253 ± 0.044 |
| Exp1-Exp4 | p<0.001 | p=0.163 ± 0.198 | p=0.006 ± 0.006 |

**Table S2.** Anova tests for the average burst inter-spike-interval curves shown in Figure 5. In cases where the curves did not differ significantly, the average p value and the respective standard deviation is reported.

| **Exp pair and Delays** | **0-90 ms** | **100-140 ms** | **150-250 ms** |
| --- | --- | --- | --- |
| Exp1-Exp2 | p=0.437 ± 0.323 | p<0.001 | p<0.001 |
| Exp3-Exp4 | p<0.001 | p=0.135 ± 0.273 | p<0.001 |
| Exp2-Exp3 | p=0.0130 ± 0.028 | p=0.279 ± 0.425 | p=0.401 ± 0.383 |
| Exp1-Exp4 | p=0.0457 ± 0.105 | p=0.279 ± 0.329 | p=0.404 ± 0.319 |

## The average burst inter-spike-interval can partially discriminate incoming signals

Synaptic clustering in the SLM layer (Figure 2C, filled red circles) resulted in slightly (~2%) larger average $ISI_{b}$ values for short delays (0-90ms) compared to the fully diffused case (Figure 3). For delays larger than 160 ms, the maximum average $ISI_{b}$ values for Exp1 and Exp3 were $24.99\pm0.31$ and $24.99ms\pm0.47$ respectively. While there was no significant difference between the average $ISI_{b}$ values of the two experiments, the two curves were fitted by sigmoid functions with different rising rates: $Rate_{Exp_{1}}\text{=14.29}ms\pm1.85$, $Rate_{Exp_{3}}\text{=18.96}ms\pm1.88$.

Synaptic clustering in the SR (Figure 2C, open red circles) was qualitatively similar to the fully clustered case (Figure 2C, black open circles). In this case the maximum average $ISI_{b_{Exp_{4}}}\text{=17.65}ms\pm0.13$ for delays between 0 and 100 ms while for delays larger than 130ms, the average $ISI_{b}$ was $21.59ms\pm0.14$. The curve was fitted by a sigmoid function with rising phase described from $Rate_{Exp_{4}}\text{=9.13}ms\pm2.01$.

## 3D return maps show that diffused inputs are more similar than clustered inputs for short delays.


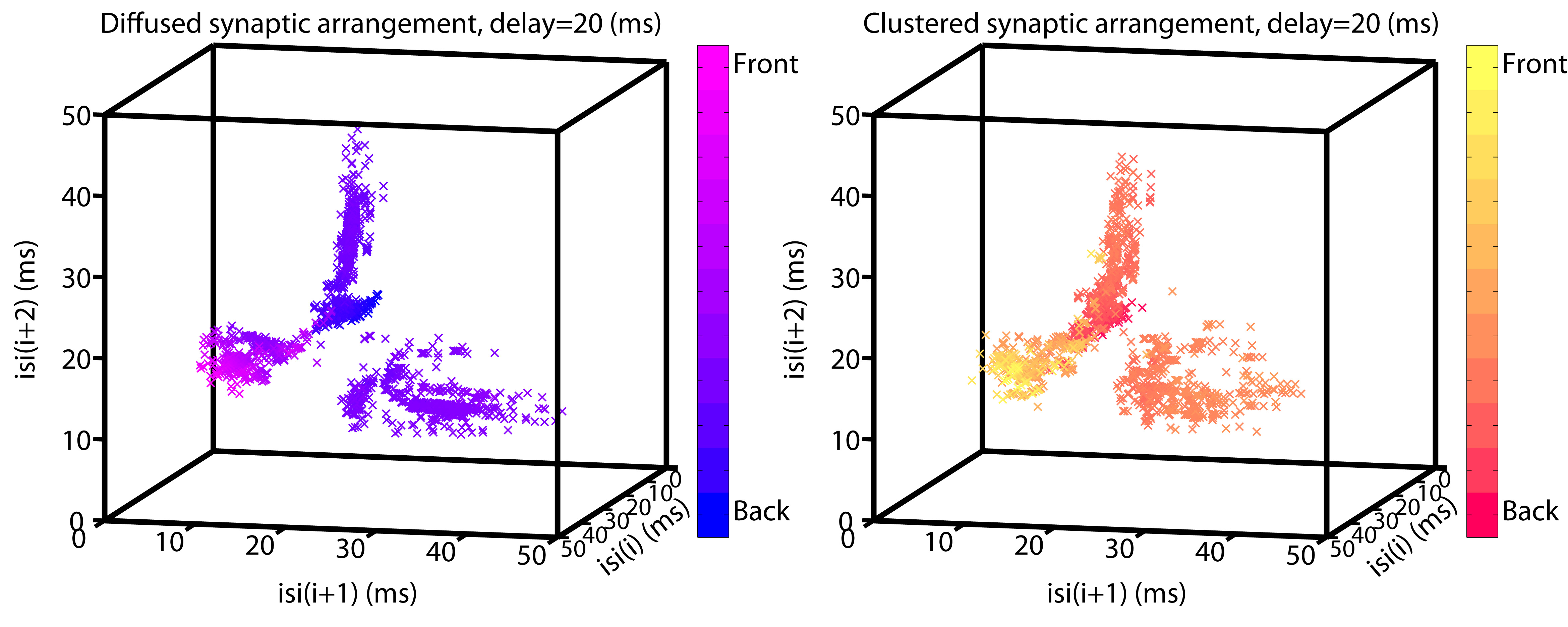


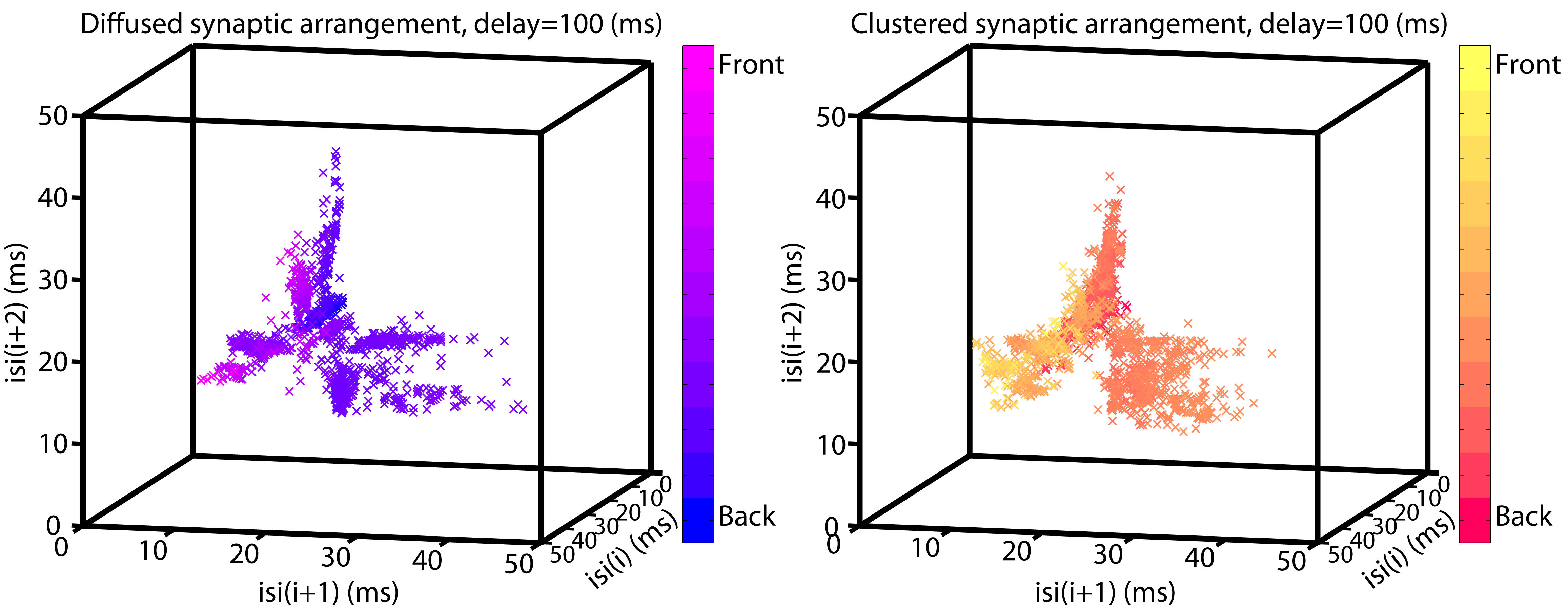


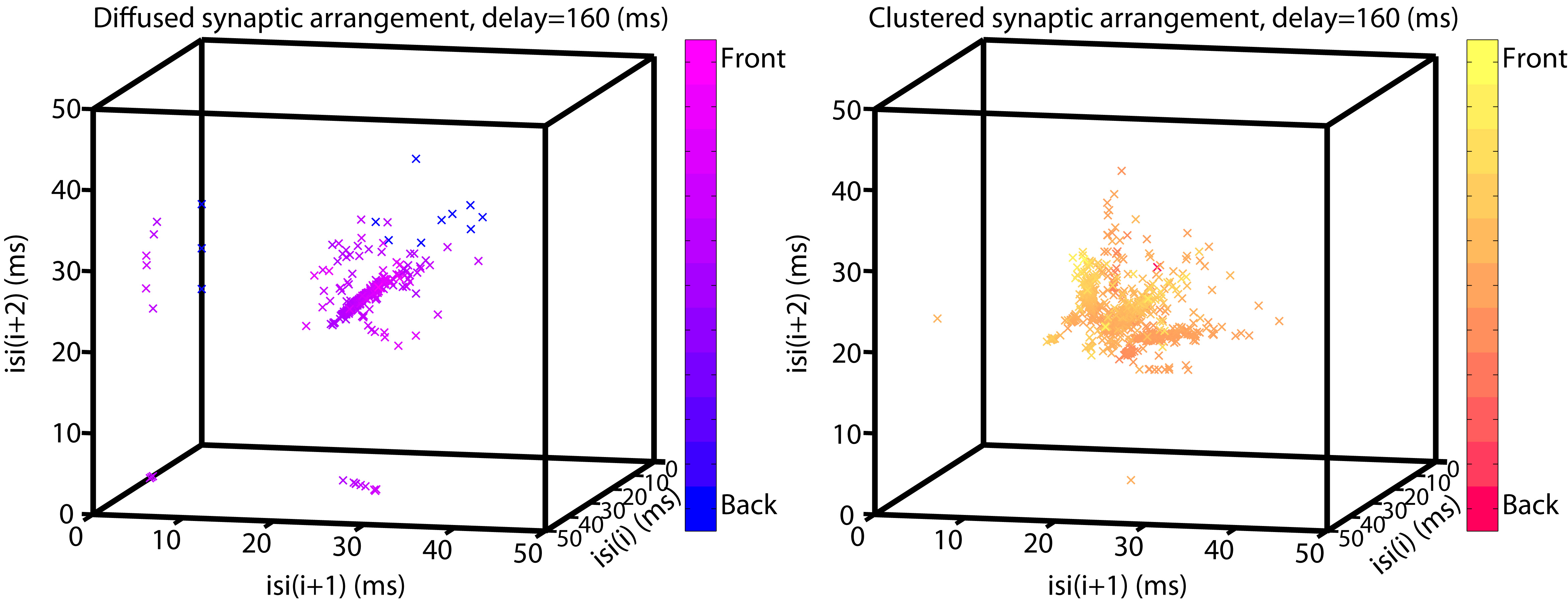


Figure S1. Three-dimensional return maps of varied firing patterns.

Fully clustered (right) and fully diffused (left) arrangements for three delays (20, 100 and 160ms). For a delay of 20ms, model responses to diffused stimuli (left) form a more structured map than responses to clustered synapses (right), whereby the ISI_b_s are condensed in several tight groups. This grouping indicates a relationship between preceding and future spike occurrences for diffused signals and appears to gradually fade in both arrangements when the temporal delay increases to 100 and 160ms.


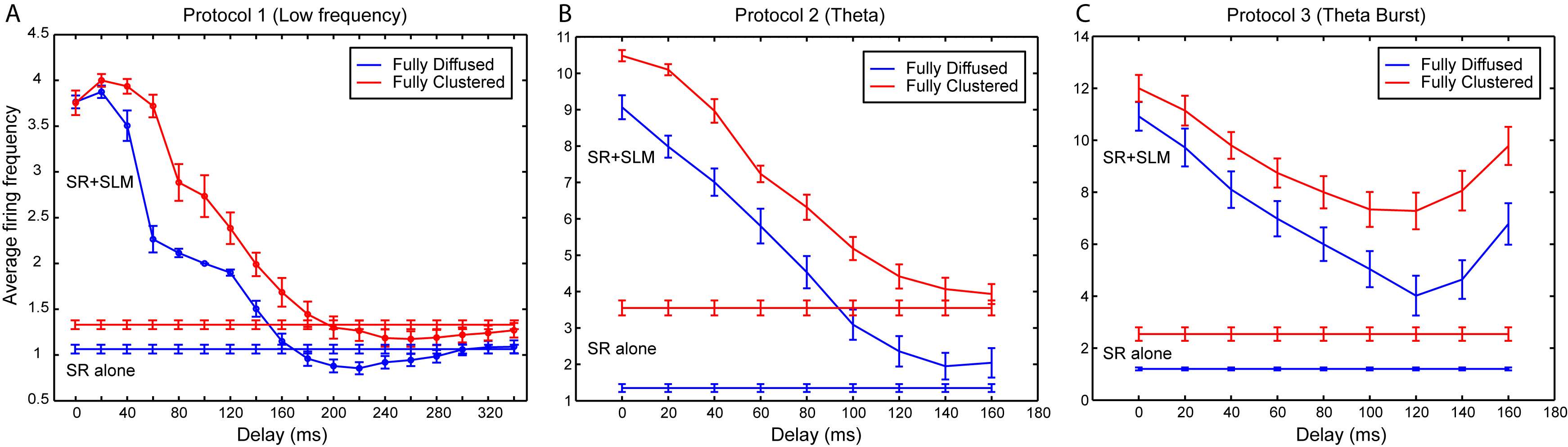


**Figure S2. Average firing frequencies of the model cell in response to three alternative stimulation protocols.** Synapses are clustered or diffused. Error bars represent standard error.

## Variability of individual neuronal responses

To examine whether the neuron is able to discriminate between different individual diffused or clustered input patterns we use the local variation *(LV)* metric described in [1]. For a given synaptic arrangement, and temporal delay each one of the 62 repetition trials is represented by a single LV number calculated over the spiking pattern produced by this trial. Figure S3 shows a K-means clustering analysis of LV values for both fully clustered and fully diffused arrangements and temporal delays equal to 0 ms and 120 ms. Distances were measured with the L1 metric and each centroid is the median of the points in the particular cluster. Silhouette function was used to determine the optimum number of clusters and distance between points in the sets was calculated using the squared Euclidean distance.

As evident from the figure, LV values in each case are organised in 2-3 large clusters, suggesting that there are at least as many different subsets of individual firing patterns that can be discriminated. To investigate the underlying mechanism leading to such a separation, we tested the hypothesis that synaptic distance from the cell body may determine the cluster to which each repetition trial will be assigned. First, we compared the distributions of synaptic distances from the soma for the 62 trials in each synaptic arrangement (every possible pair). We found that clustered arrangements had highly variable distance distributions (p < 0.05 for 47% of the comparisons) as opposed to diffused arrangements were only 3% of the distributions were different (p < 0.1, Figure S4). This was an expected result as mentioned elsewhere in this study. We then examined the distributions of distances for individual trials within each cluster. As shown in Figure S4, trials in which synapses are located closer to the soma (e.g. Experiment No 39, average synaptic distance = 542.05) have an LV value very similar the centroid of the first cluster (LV=1.37, Figure S3,A) whereas when synapses are distributed further from the soma (e.g. Experiment No 19, average synaptic distance = 613.42, LV = 1.16) they result in LV values closer to the centroid of the second cluster. Indeed, we found a negative correlation between LV and average synaptic distances for diffused arrangements for the two delays tested (r = -0.35, p < 0.05, delay = 0 ms, r = -0.32, p = 0.06, delay = 120 ms). This result did not hold for clustered arrangements; small LV values were not necessarily correlated with higher average synaptic distances (results not shown). To further investigate this phenomenon we measured the average synaptic distances from the branch point of each dendritic branch for clustered arrangements (Figure S5). LV and average synaptic distances were negatively correlated (r = -0.47, p < 0.05, delay = 0 ms) but no significant correlation was found for delay = 120 ms. We speculate than in addition to the synaptic distances within the dendritic branches other emerging behaviours implicating active conductances may account for the variability in responses for this case. Moreover, statistical comparison of LV sets for the same delay, different synaptic arrangements (Table S3) shows that the two arrangements can be discriminated based on their LV sets (p<0.05).


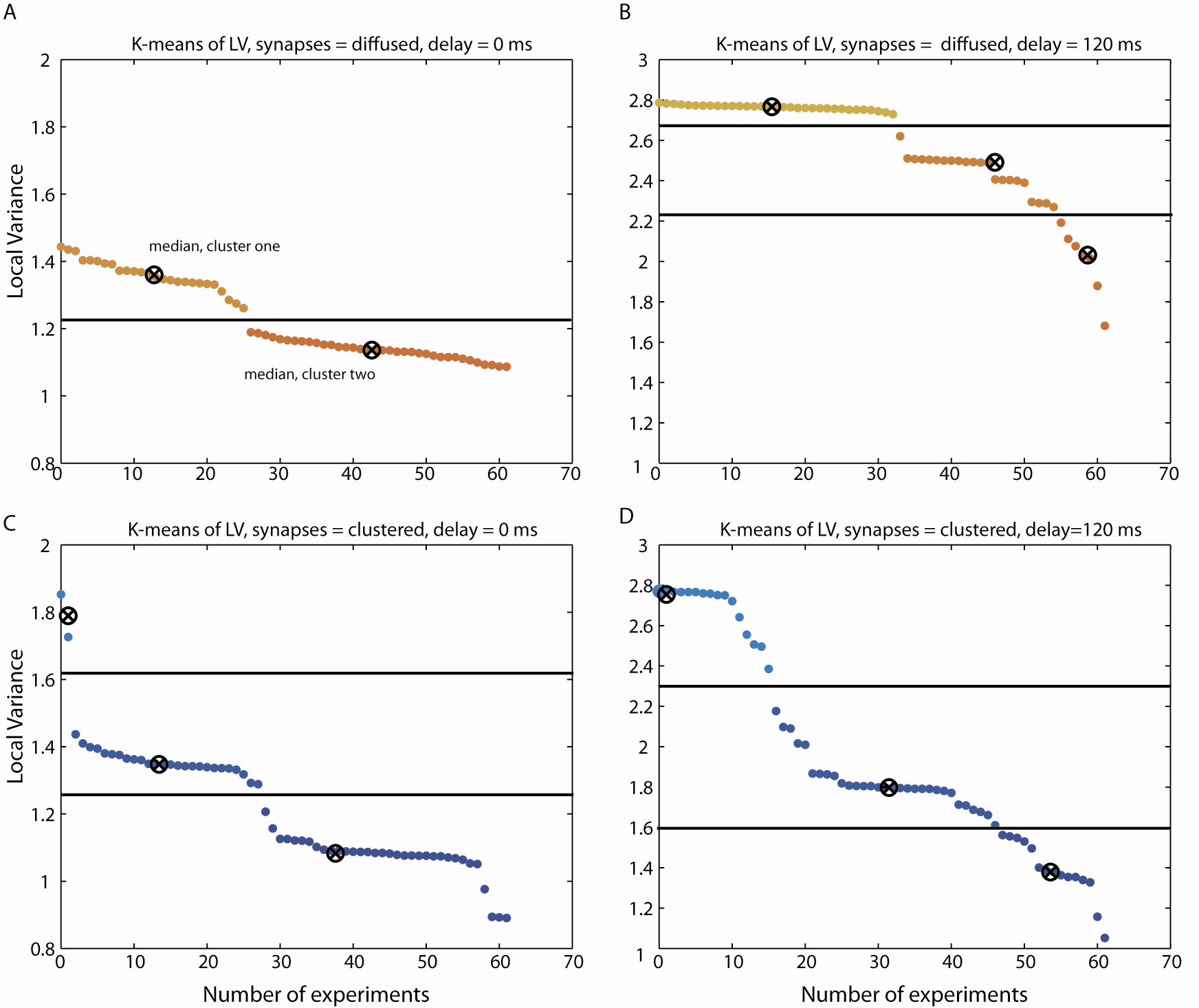


**Figure S3. Spike pattern variability assessed via the use of K-means clustering analysis.**

A, C. K-means clustering for clustered and diffused arrangements and a temporal delay = 0 ms. B, D similar for delay = 120 ms.


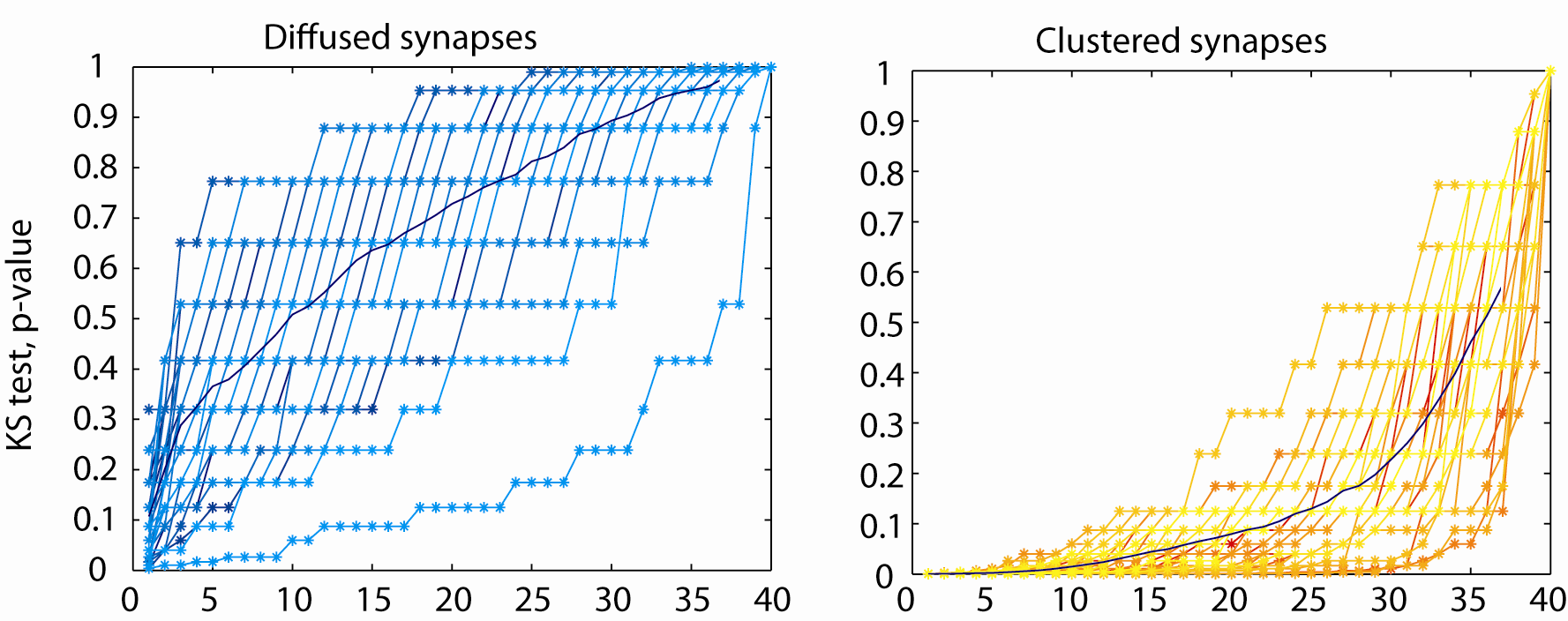


**Figure S4. Paired comparisons of the distributions of synaptic distances.**

**A, B** Differences between distributions of synaptic distances tested with two-sample Kolmogorov -Smirnov test. Each line represents the p value of one distribution compared with all the others.


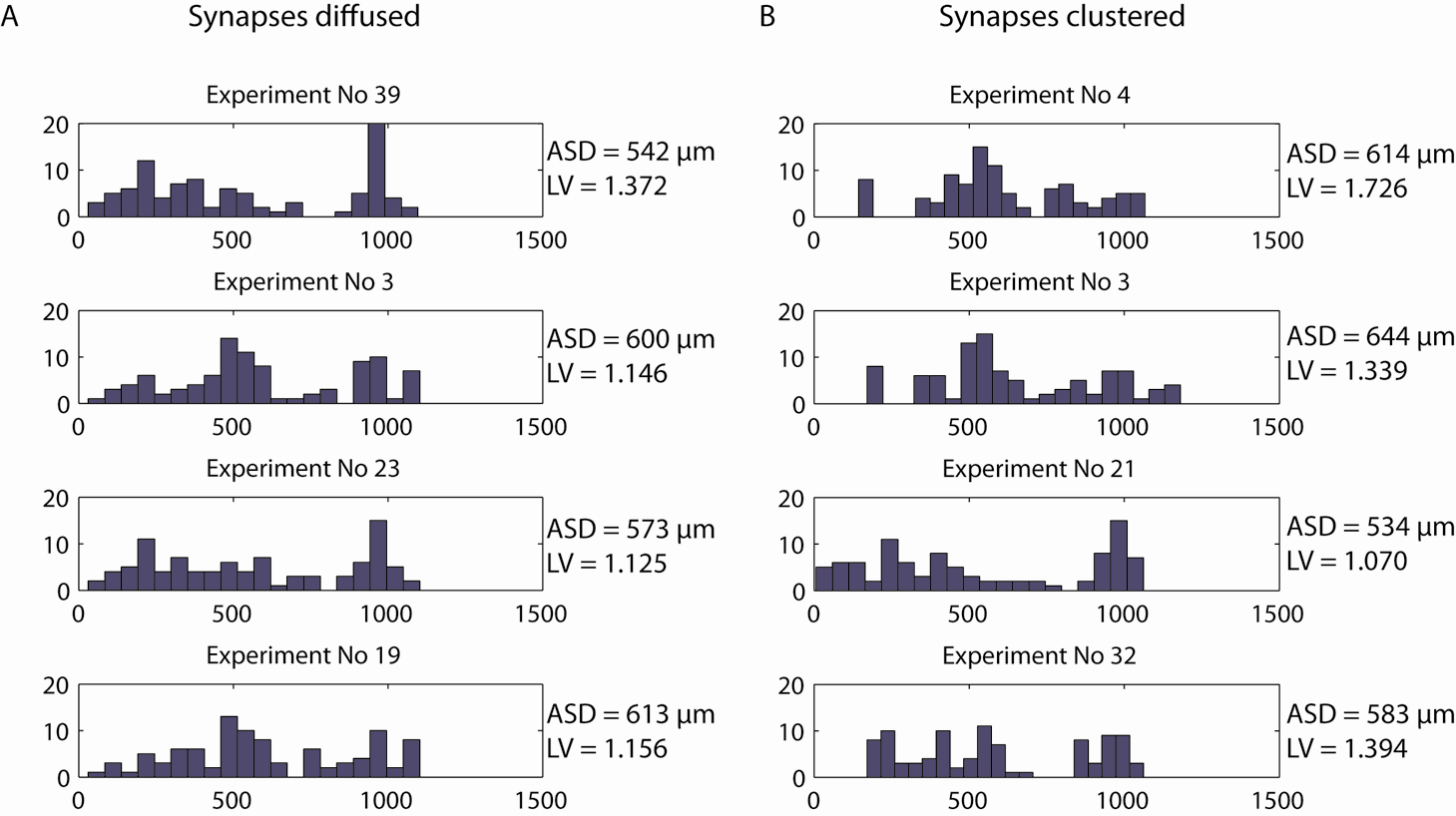


**Figure S5. Indicative histograms of synaptic distances for clustered and diffused arrangements and delay=0ms.** ASD: average synaptic distance

**Table S3.** Statistical comparison of LV values for the same delay and different synaptic arrangements. Statistical tests were performed using the Mann-Whitney U test, after Lilliefors goodness-of-fit rejected the hypothesis for normality

| Sets of local variance | p values, alpha = 0.05 |
| --- | --- |
| LV_dif_delay0 vs LV_clu_delay0 | 0.04 |
| LV_dif_delay30 vs LV_clu_delay30 | 0.0113 |
| LV_dif_delay60 vs LV_clu_delay60 | 0.0059 |
| LV_dif_delay90 vs LV_clu_delay90 | 0.0147 |
| LV_dif_delay12 0 vs LV_clu_delay120 | 7.3103e-11 |

# The model

The morphology of the CA1 model neuron is a 3D reconstruction of the n123 CA1 pyramidal cell available from <http://neuron.duke.edu/cells/index/n123_t.html>. The compartmental model is a refinement of a previously published model [2] and includes membrane and axial resistances which decrease sigmoidally with distance from soma and specific capacitance$C_{m}=\text{1μF}/cm^{2}$. It also includes 15 active conductances and four synaptic mechanisms, namely AMPA, NMDA, GABA_A_ and GABA_B_. In specific, the model includes two types of Hodgkin-Huxley sodium and potassium channels (somatic and dendritic), two A-type (I_A-prox_, I_A-dist_) and one m-type (I_M_) potassium channels, a hyperpolarization-activated nonspecific cation channel (I_h_), four voltage activated calcium currents (I_CaL_, I_CaN_, I_CaT_, I_CaR_), a calcium dependent sAHP current (I_sAHP_), a calcium dependent fAHP current (I_fAHP_) and a persistent sodium current (I_Nap_). A calcium pump/buffering mechanism is also included in the model.

## Synaptic mechanisms

### The GABA_B_ receptor

The GABA_B_ mechanism is a fundamental feature of our CA1 pyramidal neuron model. Its important contributions to long lasting inhibition and the spike blocking phenomenon has been reported in previous experimental studies [3, 4]. GABAergic neurons play a prominent role in gating the flow of information from Entorhinal cortex to CA1. Activation of the temporoammonic pathway stimulates an interneuronal network of neurogliaform interneurons, located at the borders of SR and SLM [5]. The kinetic model of GABA_B_ receptors used in this work was first described in [6]. It includes a desensitization state of the receptor and G-protein binding sites which open potassium channels. The model kinetics are given by the following equations:

$\frac{dR_{on}}{dt}=K_{1}\cdot C_{\max}\cdot(1-R_{on}-D)+K{}_{3}\cdot D$ (1)

$\frac{dR_{off}}{dt}=-K_{2}\cdot R_{off}$ (2)

$R=R_{on}+R_{off}$ (3)

$\frac{dG}{dt}=K_{3}\cdot R-K_{4}\cdot G$ (4)

$\frac{dD}{dt}=d_{1}\cdot R-d_{2}\cdot D$ (5)

$I_{GABA_{B}}=g\cdot(V-E_{rev})$ (6)

$g=\frac{G_{n}}{G_{n}+K_{D}}$, $G_{n}=G^{4}$ (7)

where the activated and deactivated states of the receptor are described by R_on_ and R_off_, respectively, G is the activated G-protein, D is the desensitized receptor and G_n_ is the n^th^ activated G-protein bound to potassium channels. The various parameter values in the above equations are listed in Table S4.

**Table S4**. Parameters of the GABA_B_ receptor model

| K_1_ (ms^-1^mM^-1^) | K_2_ (ms^-1^) | K_3_ (ms^-1^) | K_4_(ms^-1^) | d_1_ (ms^-1^) | d_2_ (ms^-1^) | K_D_ | E_rev_(mV) | n |
| --- | --- | --- | --- | --- | --- | --- | --- | --- |
| 0.52 | 0.007 | 0.058 | 0.0001 | 0.0065 | 0.000033 | 100 | -95 | 4 |

K­_1_ represents the forward binding rate, K_2_ the unbinding rate, K_3_ the rate of G-protein production unit, K_4_ the rate of G-protein decay,d_1_ the rate of desensitization, d_2_ the rate of re-sensitization and K_D_ the dissociation constant of potassium channels. Initial values for these parameters were taken from [6] and were tuned to resemble the experimental data of [7].


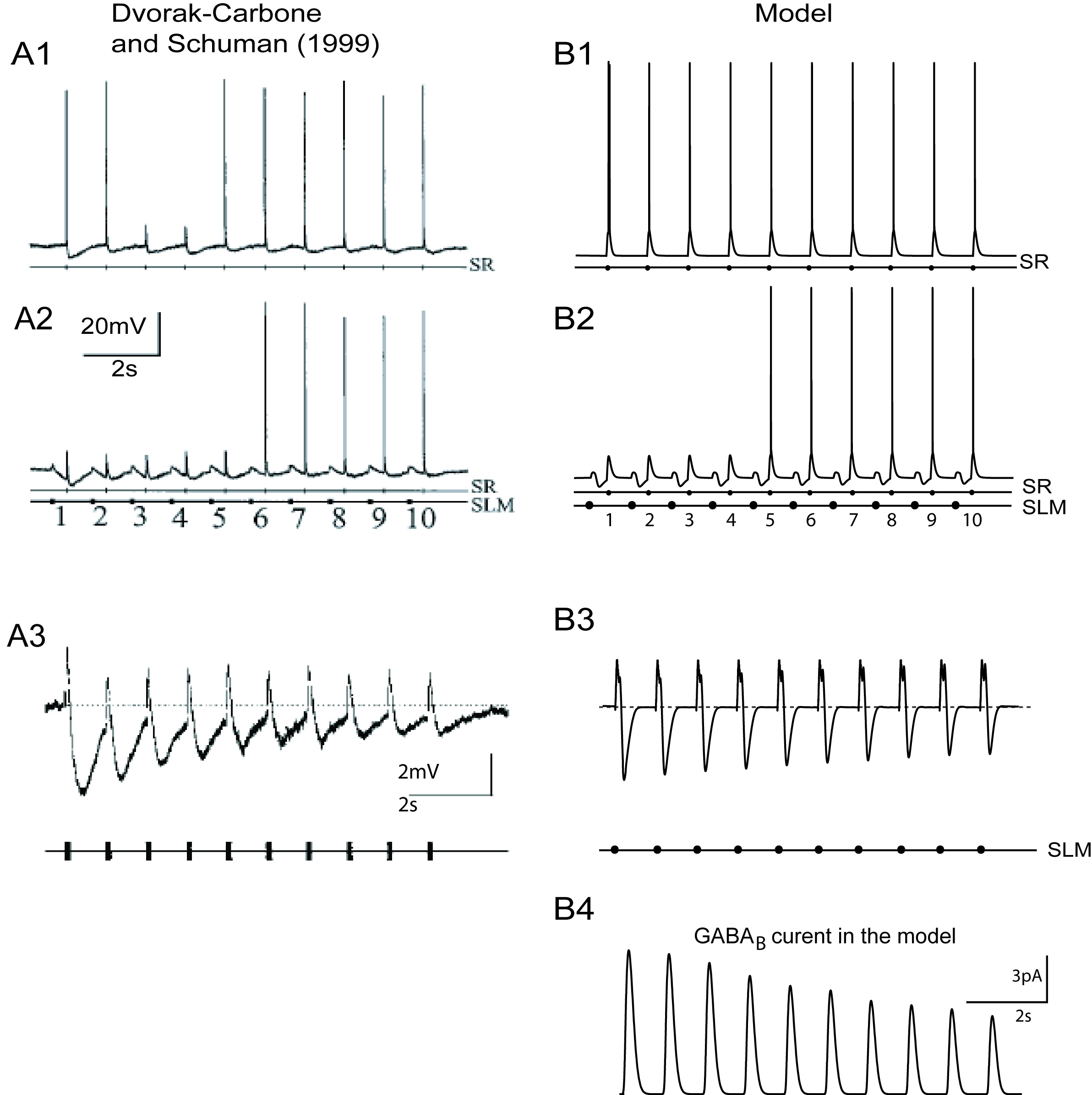


Figure S6. Experimental vs. modeling results associated with the spike blocking phenomenon and the role of GABA_B_.

A1, A2 Experimental recordings from Dvorak-Carbone and Shuman, 1999 showing somatic responses to SR stimulation (10 events at 1 Hz, A1) and SR+SLM stimulation (SLM: 10 events at 1 Hz, each event consists of 10 subthreshold pulses at 100 Hz), A2. Somatic activity is blocked for the first few spikes, when the SLM is activated 400ms prior to the SR (A2). B1, B2. Representative traces from the model for exactly the same stimulation protocol (SLM activated 400ms prior to the SR). As shown in B2, spike blocking in the model closely resembles experimental findings. A3. Experimental recordings showing somatic responses to stimulation at the SLM layer. The amplitude of the AHP following each event decreases gradually. B3. Model responses recorded at the soma for the same stimulus. A similar gradual decrease in the AHP amplitude is observed. B4. The GABA_B_ current produced by a single synapse in a distal SLM dendrite. Desensitization of the GABA_B_ receptor is evident by the decreasing amount of current along the stimulus duration

As shown in Figure S6B4, the current mediated by the GABA_B_ receptor decreases progressively with respect to the number of stimulating events, where each event is a subthreshold high frequency burst (10 pulses at 100 Hz). The figure shows a representative trace of the current produced from a single GABA_B_ receptor located at a distal dendrite of the SLM layer. The current peaks during the first event (9.2 pA) and decreases to 6.9 pA by the fifth stimulation. As shown in panel B3, activation of the synaptic mechanism at the SLM layer alone results in a biphasic response recorded at the soma: an initial subthreshold depolarization followed by a long lasting hyperpolarization, similar to the experimental data shown in panel A3.

The remaining synaptic and ionic mechanisms were not very different from the ones used in the [2,8] model. Equations describing their kinetics are listed below while more detailed descriptions can be found in the Online Supplement of the original publication.

### The NMDA receptor

$I=g\cdot(V-E_{rev})$ (4)

$g=(R_{on}+R_{off)}\cdot1(ohm^{-1})\cdot B$ (5)

$B=B(V)=\frac{1}{1+\exp(0.062(mV^{-1})\cdot(-V))\cdot(Mg/3.57(mM))}$ (6)

$\frac{dR_{on}}{dt}=\frac{synon\cdot R_{\inf}-R_{on}}{R_{\tau}}$ (7)

$\frac{dR_{off}}{dt}=-\beta\cdot R_{off}$ (8)

$R_{\inf}=\frac{C_{\max}\cdot\alpha}{C_{\max}\cdot\alpha+\beta}$ (9)

$R_{\tau}=\frac{1}{C_{\max}\cdot\alpha+\beta}$ (10)

where

$$E_{rev}=0mV,Mg=1mM,C_{\max}=1mM,\alpha=10ms^{-1}mM^{-1},\beta=0.0125ms^{-1}$$

### The AMPA receptor [9]

$I=g\cdot(V-E_{rev})$ (11)

$g=(R_{on}+R_{off})$ (12) $\frac{dR_{on}}{dt}=\frac{synon\cdot R_{\inf}-R_{on}}{R_{\tau}}$ (13)

$\frac{dR_{off}}{dt}=-\beta\cdot R_{off}$ (14)

$R_{\inf}=\frac{C_{\max}\cdot\alpha}{C_{\max}\cdot\alpha+\beta}$ (15)

$R_{\tau}=\frac{1}{C_{\max}\cdot\alpha+\beta}$ (16)

where

$$E_{rev}=0mV,C_{\max}=1mM,\alpha=10ms^{-1},\beta=0.5ms^{-1}$$

### The GABAA receptor [9]

$I_{GABA_{A}}=g\cdot(V-E_{rev})$ (17)

$g=(R_{on}+R_{off})$ (18)

$\frac{dR_{on}}{dt}=\frac{(R_{inf}-R_{on})}{R_{\tau}}$ (19)

$\frac{dR_{off}}{dt}=-\beta\cdot R_{off}$ (20)

$R_{\inf}=\frac{C_{\max}\cdot\alpha}{C_{\max}\cdot\alpha+\beta}$ (21)

$R_{\tau}=\frac{1}{C_{\max}\cdot\alpha+\beta}$ (22)

where$E_{rev}=-80mV,C_{\max}=1mM,\alpha=0.53ms^{-1}mM,\beta=0.18ms^{-1}$

## Description of ionic mechanisms

Current equations for ionic mechanisms are described by the following general equation:

$I_{i}=\overline{g}_{i}\cdot m^{M}\cdot h^{N}\cdot(V-E_{i}),$ (23)

where $\overline{g}_{i}$ is the maximum conductance of current $I_{i}$ and $E_{i}$ is the reversal potential. The variable *m* corresponds to a first order differential equation and describes the activation state of the channel. Number *M* is the number of particles required for channel activation. The variable *h* describes the inactivation state of the channel and number *N* is the number of particles required to inactivate the channel. The temperature used in this study is 34degC (celsius), Faraday constant is the magnitude of electric charge per mol of electrons, FARADAY=96485.309 C/mol, T = 273.16 in degrees Kelvin and R is the gas constant, R=8.31451 J K^-1^ mol^-1^

### The leak current

$I_{l}=g_{l}\cdot(V-E_{l})$, where *E_l_* = *-70 mV* (24)

### The fast sodium channel$I_{Na(fast)}$

$I_{Na(fast)}$ is distributed throughout the model neuron. The kinetics of the channel for all neuronal regions except the somatic and axonic compartments are given below:

$I_{Na(fast)}=\overline{g}_{Na(fast)}\cdot m^{2}\cdot h\cdot s\cdot(V-E_{Na})$ (25)

$m=\frac{m_{inf}-m}{t_{0}},t_{0}=0.05ms$ (26) $m_{inf}=\frac{1}{(1+exp((V+40)/(-3(mV))))}$ (27)

$h=\frac{h_{inf}-h}{t_{0}},t_{0}=0.5ms$ (28)

$h_{inf}=\frac{1}{(1+exp((V+45)/(3(mV))))}$ (29)

$s=\frac{s_{inf}-s}{t_{0}}$ (30)

$s_{inf}=alpha(V)+a2*(1-alpha(V))$ (31)

$alpha=\frac{1}{(1+exp((V+58)/2))}$ (32)

(32)

The time constant for the slow inactivation state (s) is given by:

$\tau=F(V)/(a0r\cdot(1+F_{1}(V)))$ (33)

where

$F(V)=\exp((10^{-3}\cdot12\cdot0.2\cdot(V+60)\cdot FARADAY)/(R\cdot(T+celsius)))$ (34)

$F_{1}(V)=\exp((10^{-3}\cdot12\cdot(V+60)\cdot FARADAY)/(R\cdot(T+celsius)))$ (35)

and a0r=0.0003 *ms^-1^*

The reversal potential for sodium is set to:

$E_{Na}$ = 50 $mV$

Channel kinetics for the axonic and somatic compartments are modified according to the following equations:

$m_{inf}=\frac{1}{(1+exp((V+44)/(-3)))}$ (36)

$h_{inf}=\frac{1}{(1+exp((V+49)/(3.5)))}$ (37)

where the Na^+^ activation time constant is now set to 0.05 ms, the inactivation time constant is set to 1 ms and the time constant for the slow inactivation state (s) remains the same.

### Slow (persistent) sodium current

Channel kinetics are given below:

$I_{Na(slow)}=\overline{g}_{Na(slow)}\cdot n^{3}\cdot(V-E_{Na})$ (38)

$n=\frac{n_{inf}-n}{t_{0}}$ (39)

$n_{inf}=\frac{1}{(1+(exp(V_{half}-V)/K))}$ (40)

where $t_{0}$ = 1 *ms*, K = 4.5, $V_{half}$ = -50.4 $mV$.

### The delayed rectifier potassium current

Channel kinetics are given below:

$I_{Kdr}=\overline{g}_{Kdr}\cdot m^{2}\cdot(V-E_{K})$ (41)

$m=\frac{m_{inf}-m}{t_{0}}$ (42)

$m_{inf}=\frac{1}{(1+exp((V+42)/(-2)))}$ (43)

$$t_{0}\text{=2.2}ms$$

At the soma, axon and dendritic trunk sections $m_{inf}$ is different:

$m_{inf}=\frac{1}{(1+exp((V+46.3)/(-3(mV))))}$ (44)

with a time constant of 3.5 *ms*

### The fast inactivating potassium current $I_{A}$

Two types of $I_{A}$ channels are included in the model neuron: The distal $I_{A_{dist}}$ and the proximal $I_{A_{prox}}$. A detailed description of the kinetics and distribution for these channels can be found in [8]. The only difference between the original and current versions of the model with respect to I_A_ is that the conductance for $I_{A_{dist}}$ is now slightly smaller: 7.2mS/cm^2^.

## Calcium currents

### *High threshold calcium current,* $I_{Ca(L)}$

The high voltage activated L-type calcium channel is colocalized with the calcium dependent potassium current responsible for the slow AHP, as described in [10]. The kinetic equations for this channel are given below:

$\overline{g}_{Ca(L)}=(\overline{g}_{Ca(L)init}\cdot m1+s^{S}\cdot8\cdot\overline{g}_{Ca(L)init})\cdot(V-E_{Ca})$ (45)

$m_{1}=m^{2}\cdot f([Ca]_{i})$ (46)

$m=\frac{m_{inf}-m}{t_{0}}$ (47)

$m_{inf}=\frac{1}{1+a}$ (48)

$a=alp(V)$ (49)

$alp(V)=\text{exp}(\frac{10^{-3}\cdot zeta\cdot(V-V_{half})\cdot FARADAY}{R\cdot(T+celsius)})$ (50)

$f([Ca]_{i})=k_{i}/(k_{i}+[Ca^{2+}]_{i})$ (51)

where $t_{0}$ = 1.5 $ms$, V_half_ = -1 *mV*, $k_{i}$= 0.025 $mM$ and $[Ca^{2+}]_{i}$the initial calcium concentration.

The current inactivates according to the state *s*, where *S=2*.

$\frac{ds}{dt}=\frac{(s_{inf}-s)}{\tau_{m}}$ (52)

$s_{inf}=\frac{alpha}{alpha+1}$ (53)

$alpha=(\frac{[Ca^{2+}]_{i}}{b})^{2}$ (54)

(55)

where b = 0.01 $mM$ and $taumin$ = 180 $ms$.

### Low threshold activated calcium current $I_{CaT}$

The kinetic equations for this channel are given below:

$I_{Ca(T)}=g_{Ca(T)_{init}}\cdot m^{2}\cdot h\cdot(V-E_{Ca})$ (56)

$\frac{dm}{d(t)}=\frac{m_{inf}-m(t,V)}{\tau_{m}(V)}$ (57)

$m_{inf}=\frac{1}{1+a}$ (58)

$a=alpm(V)$ (59)

$alpm(V)=(\frac{\text{exp}(10^{-3}\cdot zetam\cdot(V-V_{halfm})\cdot FARADAY}{R\cdot(T+celsius)})$ (60)

where$\tau_{m}$ = 1.5 $ms$ $zetam$ = -3, $V_{halfm}$ = -36 $mV$

For the inactivation state h:

$\frac{dh}{dt}=\frac{(h_{inf}-h)}{\tau_{h}}$ (61)

$h_{inf}=\frac{1}{1+b}$ (62)

$b=alph(v)$ (63)

$alph=exp(\frac{10^{-3}\cdot zetah\cdot(V-V_{halfh})\cdot FARADAY}{R\cdot(T+celsius)})$ (64)

where $\tau_{h}$ = 10$ms$ $zetah$ = 5.2, $V_{halfh}$ = -68 $mV$

### N-type Calcium current

The N-type calcium current is colocalized with the BK calcium dependent potassium current mediating the fAHP, as described in [10]

The kinetic equations for this channel are given below:

$I_{Ca(N)}=g_{Ca(N)_{init}}\cdot m^{2}\cdot h\cdot h_{2}\cdot(V-E_{Ca})$ (65)

$\frac{dm}{d(t)}=\frac{m_{inf}-m(t,V)}{\tau_{m}}$ (66)

$\frac{dh}{d(t)}=\frac{h_{inf}-h(t,V)}{\tau_{h}}$ (67)

$\frac{ds}{d(t)}=\frac{s_{inf}-s(t,V)}{\tau_{s}}$ (68)

$m_{\text{inf}}=\frac{1}{1+a}$ (69)

$a_{m}=a_{m}(V)=\text{exp}(10^{-3}\cdot z_{m}\cdot(V-V_{half_{m}})\cdot FARADAY/(R\cdot(T+celsius)))$ (70)

$h_{\text{inf}}=\frac{1}{1+b}$ (71)

$b=a_{h}(V)=\text{exp}(10^{-3}\cdot z_{h}\cdot(V-V_{half_{h}})\cdot FARADAY/(R\cdot(T+celsius)))$ (72)

$s_{\text{inf}}=\frac{a_{2}}{a_{2}+1}$ (73)

$a_{2}=(\frac{k}{[Ca^{2+}]_{i}})^{2}$ (74)

where $z_{m}=-\text{3.4,}z_{h}=\text{2, V}_{\text{half}_{m}}=-\text{21 mV, V}_{\text{half}_{h}}=-\text{40 mV, k}=\text{0.025 mM}$

### R-type calcium current [8]

The kinetic equations for this channel are given below:

$I_{CaR}=g_{CaR_{(init)}}\cdot m^{3}\cdot h\cdot(V-E_{Ca_{R}})$ (75)

$\frac{dm}{d(t)}=\frac{m_{inf}-m(t,V)}{\tau_{m}}$ (76)

$\frac{dh}{d(t)}=\frac{h_{inf}-h(t,V)}{\tau_{h}}$ (77)

$E_{Ca_{R}}=10^{3}(R\cdot(celsius+T))/(2\cdot FARADAY)\cdot\text{log}([Ca^{2+}]_{o}[Ca^{2+}]_{i})$ (78)

$m_{\text{inf}}=\frac{1}{1+\text{exp}((V+48.5)/(-3(mV)))}$ (79)

$h_{\text{inf}}=\frac{1}{1+\text{exp}((V+53)/(1(mV)))}$ (80)

where $\tau_{m}=\text{50 ms, }\tau_{h}=5ms$,

### High Threshold dendritic R-type calcium channel (CaRHd)

The kinetic equations for this channel are described in detail in [8].

## Calcium dependent potassium currents

### The fast calcium dependent potassium current $E_{K_{fAHP}}$

The channel has three states according to [11]: active (O), non active (C) and inactivated (I).

The transition from CO is calcium dependent. The transition from OI is voltage dependent and is responsible for the presence of the fast afterhyperpolarization potential. The transition from IC is voltage dependent and slow as well.

$$\begin{matrix} C\leftrightarrow O (K_{3}, K_{4}) \\ O\leftrightarrow I (K_{1}, 0) \\ I\leftrightarrow C (K_{2}, 0) \\ C+O+I=1 \end{matrix}$$

where K_1_, K_2_, K_4_ are described by function $\alpha_{2}(V)$and K_3_ by function $\alpha_{1}(V)$ and correspond to the transition rates between open (O), close (C) and inactivated (I) states:

$\alpha1(V)=\frac{1}{t_{\text{min}}+\frac{1}{\frac{1}{t_{\text{max}}-t_{\text{min}}}+e((V-V_{half})/K)\cdot1ms^{-1}}}$ (81)

(82)

Where

|  | $t_{\text{min}}$ (ms) | $t_{\text{max}}$ (ms) | $V_{half}$ (mV) | K (mV) |
| --- | --- | --- | --- | --- |
| K1 | 0.1 | - | -10 | 1.0 |
| K2 | 0.1 | - | -120 | -10.0 |
| K3 | 0.001 | 1.0 | -20 | 7.0 |
| K4 | 0.01 | - | -44 | -5.0 |

### The slow calcium dependent potassium channel $I_{K_{sAHP}}$

The kinetic equations for this channel are given below:

$I_{K(sAHP)}=\overline{g}_{sAHP}\cdot m^{2}\cdot(V-E_{K})$ (83)

$\frac{dm(t,V)}{d(t)}=\frac{m_{inf}-m(t,V)}{\tau_{m}(V)}$ (84)

$m_{inf}=\frac{a}{(a+1)}$ (85)

$a=[Ca^{2+}]/b$ (86)

$\tau_{m}=\tau_{min}+\frac{1(ms)\cdot1(mM)}{[Ca^{2+}]_{i}+b)}$ (87)

with $\tau_{min}$ = 150 $ms$ and b = 0.004 *mM*.

### Hyperpolarization Activated Current I_h_

The hyperpolarization-activated h-current maintains the same kinetics and distribution properties previously published at [8].

### Slowly Inactivating Potassium Current I_m_

The slowly inactivating potassium current I_m_ current maintains the same kinetics properties previously published in [8]. Distribution of the channel and conductance values are the same as in [10].

### D-type potassium current kdBG

The kinetic equations for the kdBG pottasium current are given below:

$I_{K}=\overline{g}_{K}\cdot m^{4}\cdot h^{4}\cdot(V+95)$ (88)

$\frac{dm}{dt}=\frac{m_{inf}-m}{\tau_{m}},\tau_{m}=1.0(ms)$ (89)

$\frac{dh}{dt}=\frac{h_{inf}-h}{\tau_{h}}$ (90)

$m_{\text{inf}}=\frac{a}{a+b}$ (91)

$a=q10\cdot\text{exp}((10^{-3})\cdot3\cdot(V-V_{half})\cdot FARADAY/(R\cdot T))$ (92)

$b=q10\cdot\text{exp}((10^{-3})\cdot(-3)\cdot(V-V_{half})\cdot FARADAY/(R\cdot T))$ (93)

$h_{\text{inf}}=\frac{a}{a+b}$ (94)

$a=q10\cdot K_{h}\cdot\text{exp}((10^{-3})\cdot(-2.5)\cdot gammah\cdot(V-V_{half_{h}})\cdot FARADAY/(R\cdot T))$ (95)

$b=q10\cdot K_{h}\cdot\text{exp}((10^{-3})\cdot(2.5)\cdot(1.0-gammah)\cdot(V-V_{half_{h}})\cdot FARADAY/(R\cdot T))$(96)

$\tau_{h}=\frac{1.0}{a+b}+\tau_{o_{h}},\tau_{o_{h}}=0$ (97)

**Table S5.** The table shows conductance and distributions of active and passive mechanisms in the various sections of the model neuron.

| **Conductances** | **Soma** | **Axon** | **Basal** | **Apical trunk** | **Oblique side branches** | | | | | |
| --- | --- | --- | --- | --- | --- | --- | --- | --- | --- | --- |
| (mS/cm^2^) |  |  | **Dendrites** |  |  | | | | | |
|  |  |  |  |  | **D*<300** | | **D>300** | | **D>350** | |
| *Nasa* | 7 | 100 | 0 | 0 | 0 | | 0 | | 0 | |
| $K_{dr}^{sa}$ | 1.4 | 20 | 0 | 0 | 0 | | 0 | | 0 | |
| *Nad* | 0 | 0 | 7 | 7 | 7 | | 7 | | 7 | |
| d | 0 | 0 | 0.867 | 0.867 | 0.867 | | 0.867 | | 0.867 | |
| *Leak=1/Rm,1/K Ωcm^2^* | 5e-3 | 5e-3 | 5e-3 | * | 6.94e-3 | | 6.94e-3 | | 6.94e-3 | |
| *Aprox* | 7.5 | 0 | 12 | ** | 0 | | 0 | | 0 | |
| *Adist* | 0 | 0 | 0 | *** | 31.245218 | | 55.6191 | | 55.6191 | |
| *h* | 1.872e-2 | 0 | 1.872e-2 | 4* | 3.04999e-2 | | 3.04999e-2 | | 3.04999e-2 | |
| *CaT* | 0.025 | 0 | 0 | 5* | 8.9693803e-2 | | 8.9693803e-2 | | 8.9693803e-2 | |
| *CaR* | 0 | 0 | 0 | 0.15 | 0.15 | | 1.95 | | 1.95 | |
| *CaLs* | 1.3851e-2 | 0 | 0 | 6* | 0 | | 0 | | 0 | |
| *CaRHd* | 0 | 0 | 0 | 7* | 3.5631e-2 | | 4.632203 | | 5.34465 | |
| *sAHP* | 0.35 | 0 | 0 | 8* | 0 | | 0 | | 0 | |
| *fAHP* | 1e-02 | 0 | 0 | 9* | 0.3 | | 0.105 | | 0.105 | |
| *M* | 2.2 | 1.1 | 0 | 2.2 | 2.2 | | 0 | | 0 | |
| *Nap* | 0 | 0 | 0 | 5.6e-04 | 2.8e-03 | | 2.8e-03 | | 8.4e-03 | |
| *KdBG* | 0.35 | 0 | 0 | 0.35 | 0.35 | | 0.35 | | 0.35 | |
| *Calcium pump* | Yes | No | No | Yes | Yes | | Yes | | Yes | |
| **Passive** |  |  |  |  |  | |  | |  | |
| **Properties** |  | |  |  | | | | | | |
| *Rm (KΩcm^2^)* | 200 | 200 | 200 | 10* | |  | |  | |  |
| *Ri (KΩcm)* | 50 | 50 | 50 | 11* | | 50 | | 50 | | 50 |
| *Cm* (*μF*/*cm^2^)* | 1 | 1 | 1 | 1 | | 1 | | 1 | | 1 |

* Leak current decreases sigmoidally from the soma to apical trunk [8]

** Linear increase along the apical trunk [8]

*** Linear increase along the apical trunk [8]

4* Sigmoidally modulated along the apical trunk [8]

5* Linear increase along the apical trunk [8]

6* If d<100, g=1.3851e-02, else g=4.1553e-02

7* If d<50, g=1.63903e-03, else g=3.5631e-02

8* If d<100 g=0.35, else g=0

9* If d<100 g=0.01, else g=0.3

10* Membrane resistance decreases sigmoidally from the soma to apical trunk [8]

11* Axial model resistance decreases sigmoidally from the soma to apical trunk [8]

where

$R_{m}(x)=R_{m_{soma}}+\frac{(R_{m_{end}}-R_{m_{soma}})}{1+\text{exp}(\frac{d_{half}-d_{x})}{steep})},steep=50microns$ (98)

$R_{i}(x)=R_{i_{soma}}+\frac{(R_{i_{end}}-R_{i_{soma}})}{1+\text{exp}(\frac{d_{half}-d_{x}}{steep})},steep=50microns$ (99)

# REFERENCES

1. Shinomoto S, Kim H, Shimokawa T, Matsuno N, Funahashi S, et al. (2009) Relating neuronal firing patterns to functional differentiation of cerebral cortex. PLoS Comput Biol 5: e1000433.

2. Poirazi P, Brannon, T. and Mel, B.W. (2003) Arithmetic of Subthreshold Synaptic Summation in a Model CA1 Pyramidal Cell. Neuron 37: 977-987.

3. Empson RM, Heinemann U (1995) Perforant path connections to area CA1 are predominantly inhibitory in the rat hippocampal-entorhinal cortex combined slice preparation. Hippocampus 5: 104-107.

4. Dvorak-Carbone H, Schuman EM (1999) Patterned activity in stratum lacunosum moleculare inhibits CA1 pyramidal neuron firing. J Neurophysiol 82: 3213-3222.

5. Price CJ, Cauli B, Kovacs ER, Kulik A, Lambolez B, et al. (2005) Neurogliaform neurons form a novel inhibitory network in the hippocampal CA1 area. J Neurosci 25: 6775-6786.

6. Destexhe A, Sejnowski TJ (1995) G protein activation kinetics and spillover of gamma-aminobutyric acid may account for differences between inhibitory responses in the hippocampus and thalamus. Proc Natl Acad Sci U S A 92: 9515-9519.

7. Dvorak-Carbone H, and Schuman, E.M. (1999) Patterned Activity in Stratum Lacunosum Moleculare Inhibits CA1 Pyramidal Neuron Firing. J Neurophysiol 82: 3213-3222.

8. Poirazi P, Brannon, T. and Mel, B.W. (2003) Online Supplement: About the Model. Neuron 37.

9. Destexhe A, Mainen ZF, Sejnowski TJ (1997) Kinetic Models of Synaptic Transmission. In: Koch C, Segev I, editors. Methods in Neuronal Modeling. 2nd ed. Cambridge, MA: MIT Press.

10. Markaki M, Orphanoudakis S, Poirazi P (2005) Modelling reduced excitability in aged CA1 neurons as a calcium-dependent process. Neurocomputing 65-66: 305-314.

11. Shao LR, Halvorsrud R, Borg-Graham L, Storm JF (1999) The role of BK-type Ca2+-dependent K+ channels in spike broadening during repetitive firing in rat hippocampal pyramidal cells. J Physiol 521 Pt 1: 135-146.
